# Effects of physical therapy modalities for early postoperative pain following total knee arthroplasty: a systematic review and network meta-analysis

**DOI:** 10.3389/fresc.2026.1780374

**Published:** 2026-06-05

**Authors:** Chao Guo, Zhen Fang, Bing Han, Yuanhao Chen, Qinghui Han, Wenbo Tang, Lujun Zhuang, Yawen Liu, Minyue Qiang, Peng Huang, Hongjing Pan

**Affiliations:** 1 School of Sports Medicine and Rehabilitation, Beijing Sport University, Beijing, China; 2 UWE College, Hainan Medical University, Haikou, China; 3 Department of Physical Education and Research, Beijing Foreign Studies University, Beijing, China

**Keywords:** network meta-analysis, physical therapy modalities, postoperative pain, rehabilitation, total knee arthroplasty

## Abstract

**Objective:**

This study aims to perform a network meta-analysis (NMA) to evaluate and compare the effects of various physical therapy modalities in mitigating early postoperative pain following total knee arthroplasty (TKA).

**Methods:**

This systematic review and NMA was conducted according to PRISMA-NMA guidelines. A comprehensive literature search was conducted across multiple databases, including PubMed, SPORTDiscus, The Cochrane Library, Embase, Web of Science, CINAHL Plus and China National Knowledge Infrastructure, to identify randomized controlled trials (RCTs) assessing the effects of eight physical therapy modalities on early postoperative pain in patients with TKA. Data were synthesized using a frequentist framework, and the outcome was pain reduction, assessed using validated pain scales such as the Visual Analogue Scale.

**Results:**

A total of 42 RCTs involving 3,165 participants were included. The NMA demonstrated that electroacupuncture, manual lymphatic drainage (MLD), kinesio taping and cryotherapy all significantly alleviated early postoperative pain following TKA compared to controls. Effect sizes [standardized mean differences (SMDs)] ranged from −0.57 [95% confidence interval (CI): −1.12 to −0.01] for MLD to −0.98 (95% CI: −1.50 to −0.45) for cryotherapy. Among these physical therapy modalities, cryotherapy showed the highest probability of being ranked among the more effective interventions (Surface under the cumulative ranking curve = 88.0%), followed by kinesio taping (83.1%) and electroacupuncture (64.0%). Among these interventions, electroacupuncture (SMD = −0.59, 95% CI: −1.19 to 0.00), kinesio taping (SMD = −0.86, 95% CI: −1.54 to −0.18) and cryotherapy (SMD = −0.95, 95% CI: −1.59 to −0.31) demonstrated significant pain reduction compared to continuous passive motion. However, the overall certainty of evidence ranged from low to very low for most comparisons.

**Conclusion:**

This NMA suggests that cryotherapy may be the most effective physical therapy modality for early postoperative pain relief after TKA, followed by kinesio taping and electroacupuncture. However, these findings should be interpreted with caution due to the overall low to very low certainty of evidence and potential risk of bias in the included studies. Further high-quality RCTs are needed to confirm these results and strengthen the evidence base for clinical decision-making.

**Systematic Review Registration:**

https://www.crd.york.ac.uk/PROSPERO/view/CRD42024518900 identifier CRD42024518900.

## 1 Introduction

Total knee arthroplasty (TKA) is one of the most common surgical procedures worldwide (1). TKA is indicated for patients with end-stage knee osteoarthritis (2), with the goals of decreasing pain, restoring mobility and function, and improving health-related quality of life (3). As the prevalence of osteoarthritis has increased, so have the number of TKAs (4). A modelling study using data from the UK National Joint Registry estimated that 186,302 patients would require primary TKA in 2030 (5). The US Nationwide Inpatient Sample estimates that 1.3 million Americans will undergo TKA in 2025, and that this figure will increase to 1.9 million by 2030 (6).

However, approximately 25% of patients report persistent postoperative pain after TKA with higher rates after revision surgery (7, 8), which remains a considerable clinical challenge. Inadequate early postoperative pain management has profound acute effects, including immune system suppression and decreased mobility. This decreased mobility, in turn, raises rates of deep vein thrombosis, pulmonary embolism, myocardial infarction, and pneumonia (9). For example, a recent retrospective cohort study reported that venous thromboembolism occurred in 1.4% of TKA patients despite perioperative pharmacological thromboprophylaxis (10), whereas imaging-based studies without prophylaxis have reported incidences as high as 69.9%, although most cases are asymptomatic (11). Long-term influences of poor pain management include transition to chronic pain and prolonged narcotic consumption (12). These outcomes can impede rehabilitation, delay patient recovery and increase the incidence of immobility-related complications (13), thereby leading to a longer hospital stay and higher expenses (14, 15), which will greatly increase the burden on individuals and society. Therefore, ensuring effective and timely pain management throughout the treatment process is essential.

First-line therapies to treat postoperative pain are pharmacological, including anesthetics, opioids and acetaminophen (13, 14). However, the analgesic benefits of these agents are often modest. Effect sizes for nonsteroidal anti-inflammatory drugs (NSAIDs) and opioids frequently fall below the minimal clinically important difference (MCID) for pain improvement in musculoskeletal conditions (16). Moreover, the regular use of pharmacological agents for pain may be associated with significant side effects (17). For example, clinical upper gastrointestinal events may occur in 3%–4.5% of patients taking NSAIDs, and serious complicated events develop in approximately 1.5% (18). Opioid therapy also carries substantial risks, including nausea, constipation, delirium, and dependence (19, 20). As a result, many patients seek nonpharmacological alternatives for pain relief (21). Accordingly, identifying effective nonpharmacological strategies for postoperative pain management has become a key scientific priority.

Recently, nonpharmacological approaches to pain management aimed at reducing the use of prescription medications have increased (22). In clinical practice, numerous physical therapy modalities, which may have low adverse effects and are also very accessible in clinical practice, have been employed for pain management following TKA, with multiple studies evaluating their effectiveness. Yuksel et al. (23) reported that a significant difference was determined in terms of pain in kinesio taping group compared to the control group at discharge, but there was no difference between kinesio taping group and cryotherapy group in terms of pain at discharge. Guney-Deniz et al. (24) observed that both manual lymphatic drainage (MLD) and kinesio taping improved pain levels at the early stage following TKA and comparable effects between MLD and kinesio taping. Furthermore, evidence regarding the effects of these physical therapy modalities for pain management remains conflicting. For example, while one study (25) reported that continuous passive motion (CPM) provided clinically significant pain relief during postoperative rehabilitation, several other studies (25–27) have reported no significant pain reduction with CPM use. Despite the growing use of various physical therapy modalities in clinical practice, their comparative effectiveness remains uncertain. Existing studies often yield inconsistent results, and the lack of direct head-to-head comparisons between different modalities further complicates clinical decision-making. Consequently, it remains unclear which physical therapy modality is most effective for reducing postoperative pain in patients undergoing TKA.

Several meta-analyses have been conducted to compare the effects of physical therapy modalities for pain management following TKA (28–31). However, traditional meta-analysis can only compare differences in efficacy between two interventions and is insufficient when comparing three or more interventions. Furthermore, the lack of head-to-head randomized controlled trials (RCTs) in many interventions challenges the traditional evidence synthesis method of a two-arm meta-analysis (32). Network meta-analysis (NMA) extends the concept of the traditional meta-analysis to produce pairwise comparisons and relative treatment effects across a range of interventions through direct and indirect comparisons (33). The method allows comparisons among treatment modalities that have never been directly compared, increases precision, and provides a relative ranking of all modalities while properly accounting for correlations between effect sizes from multi-arm trials (30, 31). Therefore, we conducted a NMA to compare the effects of various physical therapy modalities for early postoperative pain management following TKA, aiming to identify the most effective physical therapy modality.

## 2 Methods

This systematic review and NMA was registered with PROSPERO (CRD 42024518900) and conducted following the guidelines outlined in the Preferred Reporting Items for Systematic Reviews and Network Meta-Analyses (PRISMA-NMA) statement (34). The PRISMA checklist is available in Supplementary Table S1.

### 2.1 Search strategy

The databases for PubMed, SPORTDiscus, The Cochrane Library, Embase, Web of Science, CINAHL Plus and China National Knowledge Infrastructure (CNKI) were searched online from inception to 1 June 2024. The search strategy was developed using a combination of relevant free text terms, synonyms and subject headings such as total knee arthroplasty, physical therapy modalities, pain and randomized controlled trial. Boolean logical operators were used to connect the search terms. The search strategy was tailored for each database according to its specific requirements and was reviewed by a medical librarian and by physical therapy experts to ensure its comprehensiveness and accuracy. Detailed search strategies for each database are presented in Supplementary Table S2. To avoid missing eligible literature, we cross-checked the reference lists of pairwise meta-analyses on related topics published in the past decade.

### 2.2 Study selection and eligibility criteria

Duplicates were first removed using Zotero software. To avoid missing eligible literature, we performed a manual check after removing duplicates. Two researchers (CG and YHC) independently screened the titles, abstracts and full texts for inclusion.

To assess the interrater reliability between the 2 independent reviewers, the Cohen kappa statistic (*κ*) was calculated, yielding a value of 0.81 for title/abstract screening and 0.84 for full-text screening, indicating almost perfect agreement. If there was any disagreement in study selection between the two researchers, it was resolved by discussion or a third researcher (BH).

We employed the PICOS (Population-Intervention-Comparison-Outcome-Study Design) framework to screen articles for inclusion. The detailed inclusion criteria were as follows: (1) Participants (P): Patients operated with primary TKA for knee osteoarthritis, ≥18 years old; (2) Intervention (I): The experimental group received one physical therapy modality in addition to standardized postoperative care (e.g., analgesics, education, muscle-strengthening exercises, functional exercises, gait training and immobilization); (3) Comparison (C): The control group did not receive physical therapy modality or received a corresponding placebo intervention, with standardized postoperative care provided equally to both groups; (4) Outcomes (O): Pain assessed by a clinically validated scale, such as visual analogue scale (VAS), Numerical Rating Scale (NRS), McGill Pain Questionnaire and pain subscale of Western Ontario and McMaster University Osteoarthritis index (WOMAC) or pain subscale of Knee Society Score (KSS); and (5) Study Design (S): Only RCTs were included. The study languages were restricted to English and Chinese.

The exclusion criteria were as follows: (1) Non-RCTs; (2) Patients operated by TKA for rheumatic arthritis or tumors around the knee or did not undergo knee arthroplasty; (3) Studies that did not report relevant clinical outcomes; (4) Duplicate publications or studies with overlapping data; and (5) Incomplete, ambiguous or non-extractable data. When multiple articles were from the same study and reported the same or overlapping results, we included only the most recently published article. During the screening process, if a type of physical therapy modality appeared in two or more studies, it was considered for inclusion.

### 2.3 Outcomes

The outcome in this study was postoperative pain scores, assessed using VAS, NRS, McGill Pain Questionnaire, pain subscales of WOMAC or KSS. When multiple instruments were reported within a single trial, we applied a predefined hierarchy to ensure consistency and reduce selection bias. Specifically, the VAS was prioritized given its widespread use and validity in TKA studies, followed by the NRS, the McGill Pain Questionnaire, and pain subscales of WOMAC and KSS. Mean changes in pain scores were calculated using the reported means (M), standard deviations (SD) and sample sizes. If a study did not report changes in M and SD, the following formulas were used to calculate the change: M (change) = M (final) − M (baseline), and SD(change)= $\sqrt{{\left(SD_{\mathrm{baseline}}\right)}^{2}+{\left(SD_{\mathrm{final}}\right)}^{2}-{\left(2\times r\times SD_{\mathrm{baseline}}\times SD_{\mathrm{final}}\right)}^{2}}$. In this formula, r is the pre-post test correlation coefficient. Based on the Cochrane Handbook's suggestion, we adopted a value of r = 0.50 for the correlation coefficient in the main analysis. Since r were rarely reported in the included studies, we derived values ranging from r = 0.41 to 0.79 (35–37) based on the available study data and in accordance with the manual's recommendations. Consequently, we conducted a sensitivity analysis by re-specifying the value of r as 0.75 and re-running the NMA.

The period within 0–2 weeks after TKA is generally regarded as the early postoperative phase (38), during which pain levels are typically highest and recovery interventions are most critical. In order to observe the effects of physical therapy modalities on early postoperative pain following TKA, we extracted baseline pain scores prior to the intervention and postoperative pain scores within 2 weeks after the intervention.

### 2.4 Data extraction

Data extraction was conducted independently by two researchers (CG and YHC). Disagreements were resolved by discussion or a third researcher (BH). The extracted data included study characteristics (first author, publication year, country and sample size per group), participants’ mean age, sex, sex ratio, intervention parameters (type, frequency, intensity, duration per session and length of intervention), comparator information, tools used to assess outcomes and outcomes (M ± SD). In cases of incomplete data, we attempted to contact the corresponding author via email for clarification.

### 2.5 Risk of bias and certainty assessment

The risk of bias was independently assessed by two researchers (QHH and YHC), with any disagreements resolved by a third researcher (BH). The assessment was conducted at the outcome level, specifically for pain outcomes, using the Cochrane Risk of Bias 2.0 tool (39). Each trial was evaluated across five domains: (1) randomization process; (2) deviations from intended interventions; (3) missing outcome data; (4) measurement of the outcome; and (5) selection of the reported result. Judgments were made separately for the pain outcome, which was the predefined primary endpoint of this study. Regarding the effect of interest, pain outcomes were extracted according to the intention-to-treat (ITT) principle whenever available; when trials did not explicitly report ITT analyses, we used the results as presented, which in most cases reflected an ITT approach given the minimal attrition observed during the 0–2 weeks postoperative period. The risk-of-bias assessment and effect estimation were therefore based on this early postoperative phase (0–2 weeks), consistent with our predefined outcome window.

We used the Confidence in Network Meta-Analysis (CINeMA) web tool (40) to assess the quality of evidence for each comparison. Our approach was guided by practices established in previous literature (41, 42). The tool considers six factors that could lead to a downgrade: within-study bias, reporting bias, indirectness, imprecision, heterogeneity and incoherence. For specific operational methods and definitions, refer to Supplementary Table S3.

### 2.6 Statistical analysis

NMA was performed according to the current PRISMA NMA guidelines (43), employing a frequentist framework using STATA 17.0 software. Since outcomes in this meta-analysis are continuous, effect sizes are expressed as standardized mean differences (SMDs) with 95% confidence intervals (CIs) for outcomes measured with varying instruments. The NMA procedure consisted of the following steps: (1) A network geometry was created to explore the comparative relationships between several interventions and controls. (2) Inconsistency tests were performed. The node-splitting method was used to assess the consistency between direct and indirect evidence within that loop. (3) A league table was generated to depict the pairwise comparative effects of the physical therapy modalities. This table offered a clear and concise representation of the efficacy of each treatment. (4) The interventions were ranked to determine their superiority based on the surface under the cumulative ranking curve (SUCRA) and mean rank. The SUCRA rank-probability curve was plotted; the larger the area under the curve or SUCRA value (0%–100%), the more effective intervention was in alleviating pain (44). (5) Network funnel plots were visually inspected using symmetry criteria to check for publication bias in the NMA. SMD were classified as trivial (<0.2), small (0.2–0.5), medium (>0.5–0.8), and large (>0.8) (45). The STATA code used for the NMA is provided in Supplementary Table S4.

### 2.7 Assessing the assumption of transitivity and consistency

The transitivity assumption is a fundamental requirement for the validity of NMA. It requires that the different sets of RCT are similar, on average, in all important factors other than the intervention comparison being made. With reference to previous studies (43, 46) and the Cochrane Handbook (39), we evaluated and supported transitivity assumption through the following approaches: (1) implementing strict selection criteria for study populations and conditions to minimize clinical and methodological heterogeneity among included trials; (2) providing detailed descriptions of participant and intervention characteristics across studies; (3) visualizing publication year, participant age, baseline pain intensity (0–100 scale), and sample size using boxplots—stratified by intervention (node-level) and comparison (edge-level)—to visually assess the distribution and balance of these potential effect modifiers across treatment nodes and comparisons; (4) conducting network meta-regression to examine whether publication year, baseline pain intensity, or intervention parameters (including number of sessions, timing of initiation, and duration) acted as effect modifiers.

Consistency, also known as coherence, serves as an empirical measure of transitivity. It implies an agreement between direct and indirect evidence for any given treatment comparison (39). We performed global and local inconsistency tests to statistically evaluate whether NMA's consistency assumptions are met. The design-by-treatment interaction model was used to assess global inconsistency and the node-splitting analysis was used to assess local inconsistency.

## 3 Results

### 3.1 Literature selection

**Figure 1:**
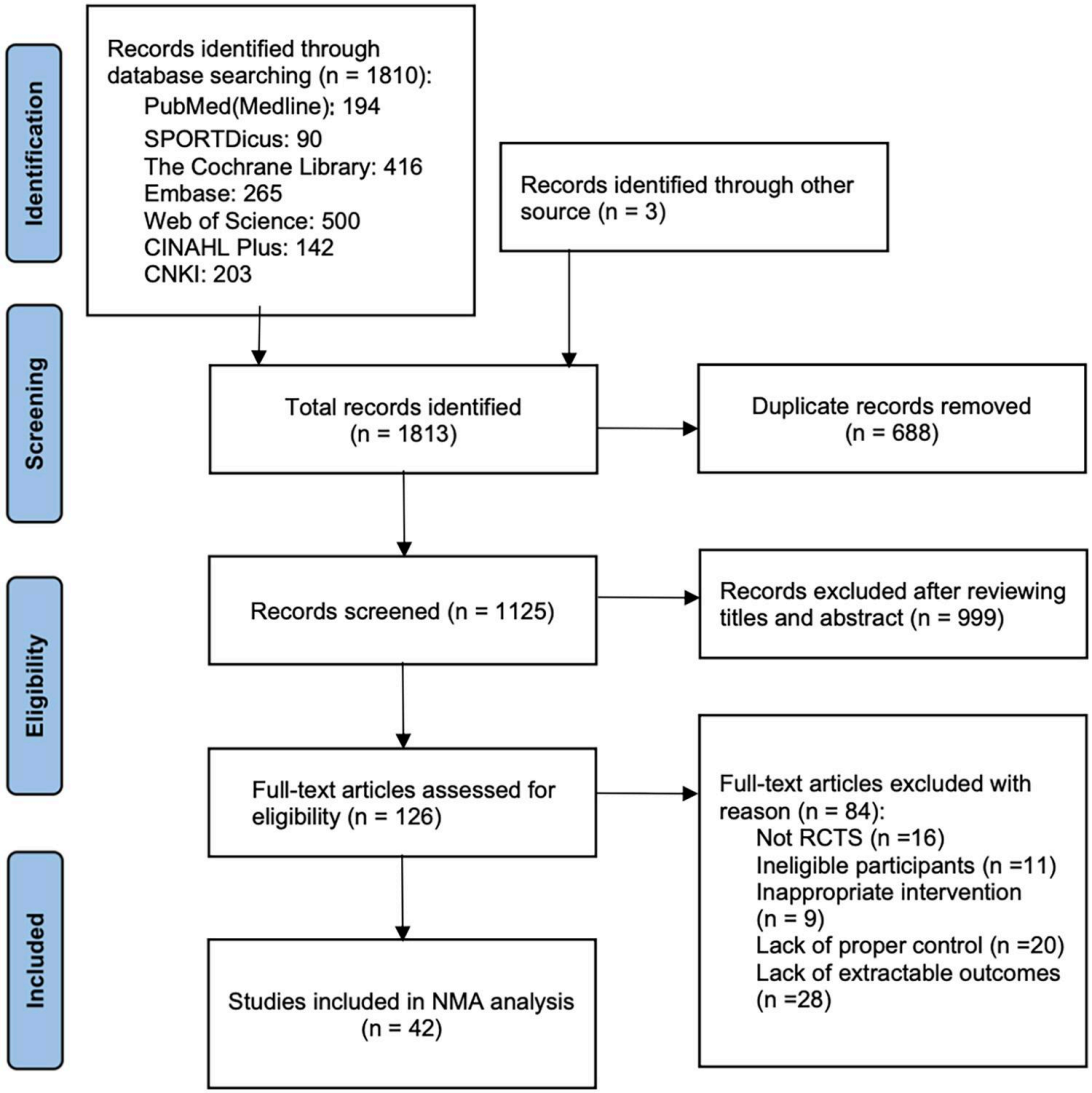
Flowchart of the screening process.

A total of 1,810 articles were identified through the search strategy, with an additional three articles identified from the reference lists of previous pairwise meta-analyses. After removing 688 duplicates using Zotero, 1,125 unique records remained. Of these studies, 126 were deemed potentially relevant following an initial screening of titles and abstracts. Subsequently, 84 studies were excluded according to this study's inclusion and exclusion criteria after full text reviewing. Of these 84 studies, 16 were excluded for not being RCTs, 11 due to ineligible participants, 9 for inappropriate intervention, 20 for lack of proper control, and 28 for lack of extractable outcomes. Description of studies excluded are provided in Supplementary Table S5. Finally, 42 studies were included in this NMA. The detailed study selection process is illustrated in Figure 1.

### 3.2 Studies' characteristics

The quantitative analysis included data from 3,165 participants (54.03% female; 1,704 in the treatment group and 1,461 in the control group) with mean age ranging from 47.4–76 years across 42 RCTs. Baseline pain intensity was consistently moderate to severe, with mean VAS scores (0–10) typically ranging from 6 to 8. Intervention parameters (e.g., frequency, duration, intensity) showed expected variability across studies but were sufficiently consistent within each physical therapy modality. For instance, cryotherapy commonly involved intermittent application (20–30 min every 2–4 h) following TKA. All trials provided standardized postoperative care to both intervention and control groups, ensuring a stable background therapy. This universally included multimodal pharmacologic analgesia, primarily NSAIDs and/or opioids, complemented by essential rehabilitation exercises. Inspect transitivity visually by creating box plots depicting effect modifiers for each treatment node and corresponding pairwise comparisons, refer to Supplementary Figure S1. In our study, inspection of the boxplots for publication year, participant age, baseline pain intensity (0–100 scale), and sample size for treatment nodes and pairwise comparisons mostly suggest that the transitivity assumption holds. The included RCTs, published between 1996 and 2023, were conducted across 14 countries: 23 from China (54.8%), four from Turkey (9.5%), two each from America, Sweden and Japan (4.8%), and one each from Belgium, Norway, India, Australia, Spain, Netherlands, Switzerland, Greece and Italy (2.4%).

The included RCTs evaluated the effects of eight distinct physical therapy modalities: three (85 participants) investigated acupuncture (47–49), 11 (376 participants) examined electroacupuncture (50–60), two (55 participants) examined the transcutaneous electrical nerve stimulation (TENS) (61, 62), four (120 participants) examined the neuromuscular electrical stimulation (NMES) (36, 38, 63, 64), six (154 participants) focused on MLD (24, 37, 51, 65–67), six (249 participants) explored kinesio taping (23, 24, 37, 68–70), six (278 participants) analyzed cryotherapy (23, 61, 62, 71–73), nine (387 participants) evaluated CPM (25–27, 35, 73–77). Of these RCTs, 37 compared the effects of physical therapy modalities with control groups, five compared two different physical therapy modalities and two employed a three-arm design. All included studies reported pain outcomes, with 33 studies using VAS, seven employing NRS and two utilizing the pain subscale of KSS. Detailed characteristics of the included studies are provided in Supplementary Table S6. It should be noted that some physical therapy modalities, including TENS, NMES, and acupuncture, were supported by a relatively small number of studies, which may limit the robustness and precision of the corresponding estimates.

### 3.3 Risk of bias and certainty assessment

**Figure 2:**
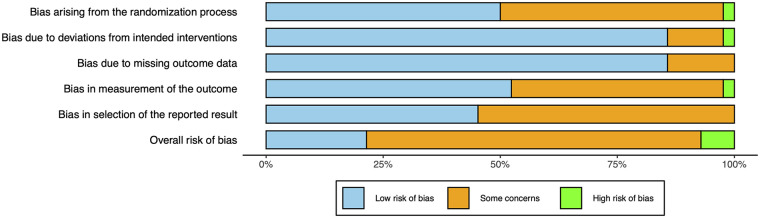
Risk of bias chart of randomized controlled trials included in the quantitative analysis.

Figure 2 provides a summary of the risk of bias assessment for the included RCTs. The majority were rated as having some concerns (*n* = 30, 71.4%), followed by low risk (*n* = 9, 21.4%) and high risk (*n* = 3, 7.2%). The results for each risk of bias indicator were as follows (low risk, some concerns and high risk): (1) randomization process (50%, 47.6% and 2.4%, respectively); (2) deviations from intended interventions (85.7%, 11.9% and 2.4%, respectively); (3) missing outcome data (85.7%, 14.3% and 0%, respectively); (4) measurement of the outcome (52.4%, 45.2% and 2.4%, respectively); and (5) selection of the reported result (21.4%, 71.4% and 7.1%, respectively). Overall, the risk of bias was considered acceptable in most RCTs. Detailed information about the risks of bias for each RCT is provided in Supplementary Figure S2.

The strength of evidence for each network estimate was provided in Supplementary Table S3. Overall, the comparisons involved in the NMA showed low or very low (for the vast majority of comparisons) quality of evidence. The main reasons for downgrading were within-study bias and reporting bias, primarily due to the high risk of bias and the limited number of studies (as assessing publication bias typically requires more than 10 studies, a condition that is often difficult to meet). In the dimensions of Indirectness and Incoherence, all comparisons demonstrated a high level of evidence certainty.

### 3.4 Outcomes of network meta-analysis

**Figure 3:**
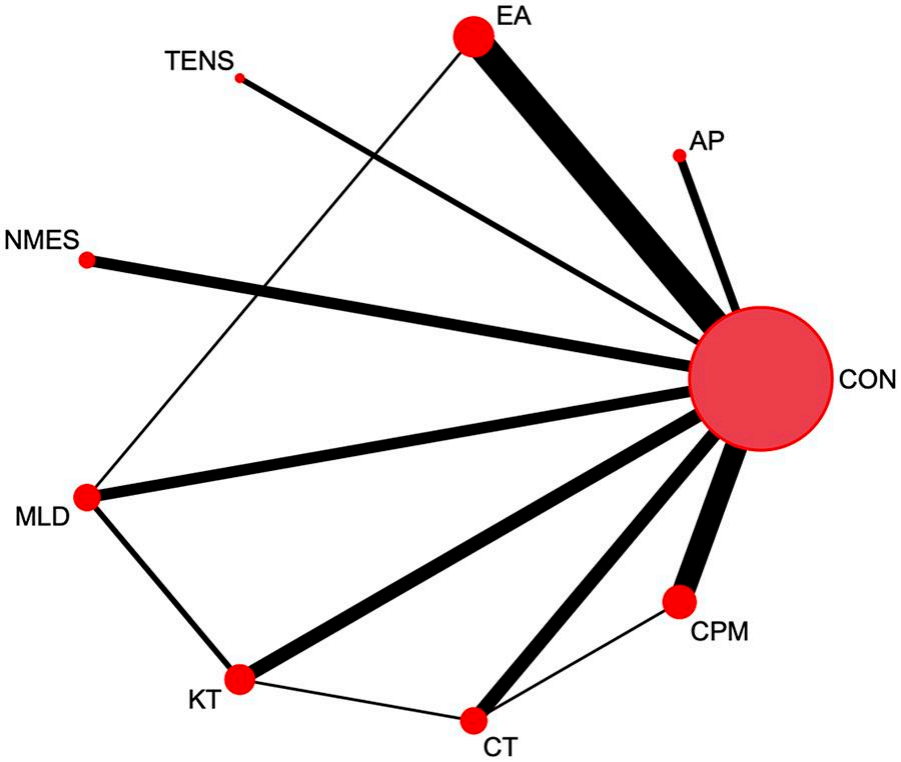
Network plot of comparisons for pain in the network meta-analysis. CON, control; AP, acupuncture; EA, electroacupuncture; TENS, transcutaneous electrical nerve stimulation; NMES, neuromuscular electrical stimulation; MLD, manual lymphatic drainage; KT, kinesio taping; CT, cryotherapy; CPM, continuous passive motion.

This NMA included 42 studies with a total of 3,165 participants, evaluating the effects of eight physical therapy modalities on early postoperative pain following TKA. Detailed definition of physical therapy modalities included is provided in Supplementary Table S7. The network plot (Figure 3) illustrates direct comparisons among the physical therapy modalities. The global inconsistency test indicated no significant inconsistency in the results (*p* > 0.05). Furthermore, the node-splitting analysis showed no significant inconsistency (*p* > 0.05; Supplementary Figure S3). These findings indicate a high level of consistency between direct and indirect evidence across both global and specific comparisons. In the network plot, each node represents a physical therapy modality, with the node size proportional to the number of participants receiving that physical therapy modality. The lines connecting the nodes represent direct comparisons, with their thickness proportional to the number of studies comparing the two interventions.

**Figure 4:**
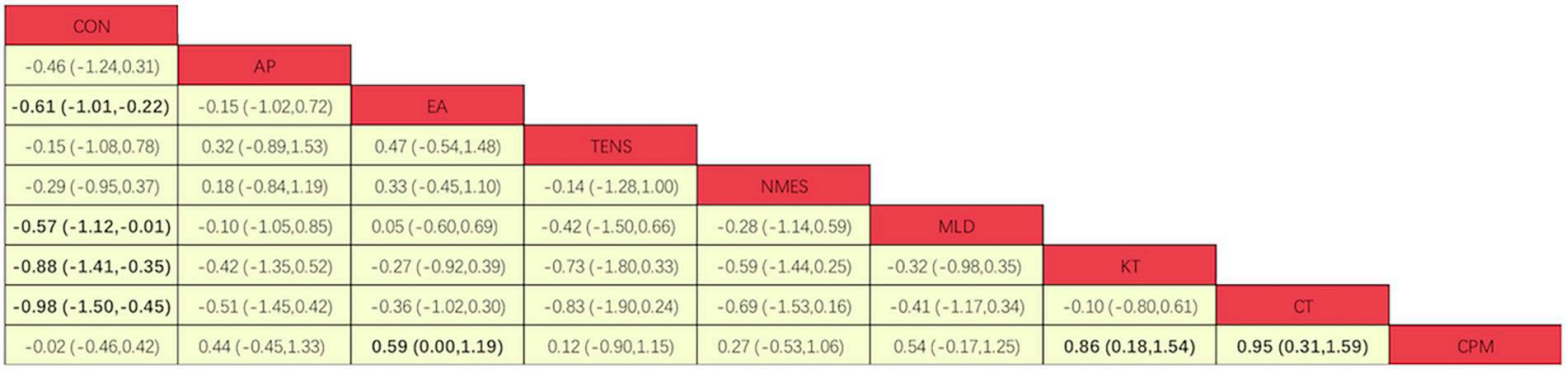
Network meta-analysis matrix of results for pain. CON, control; AP, acupuncture; EA, electroacupuncture; TENS, transcutaneous electrical nerve stimulation; NMES, neuromuscular electrical stimulation; MLD, manual lymphatic drainage; KT, kinesio taping; CT, cryotherapy; CPM, continuous passive motion.

The comparative results among interventions are presented in Figure 4. For the prediction interval plot, refer to Supplementary Figure S4. The NMA results indicated that, compared to the control group, electroacupuncture, MLD, kinesio taping and cryotherapy significantly reduced early postoperative pain following TKA, with effect sizes (SMDs) ranging from −0.57 (95% CI: −1.12 to −0.01) for MLD to −0.98 (95% CI: −1.50 to −0.45) for cryotherapy. Among the physical therapy modalities, electroacupuncture (SMD = −0.59, 95% CI: −1.19 to 0.00), kinesio taping (SMD = −0.86, 95% CI: −1.54 to −0.18) and cryotherapy (SMD = −0.95, 95% CI: −1.59 to −0.31) demonstrated significant pain reduction compared with CPM. No significant differences were identified among the other physical therapy modalities according to the league tables. However, for some interventions such as TENS, NMES, and acupuncture, the available evidence was based on a limited number of studies, and therefore these results should be interpreted with caution.

**Figure 5:**
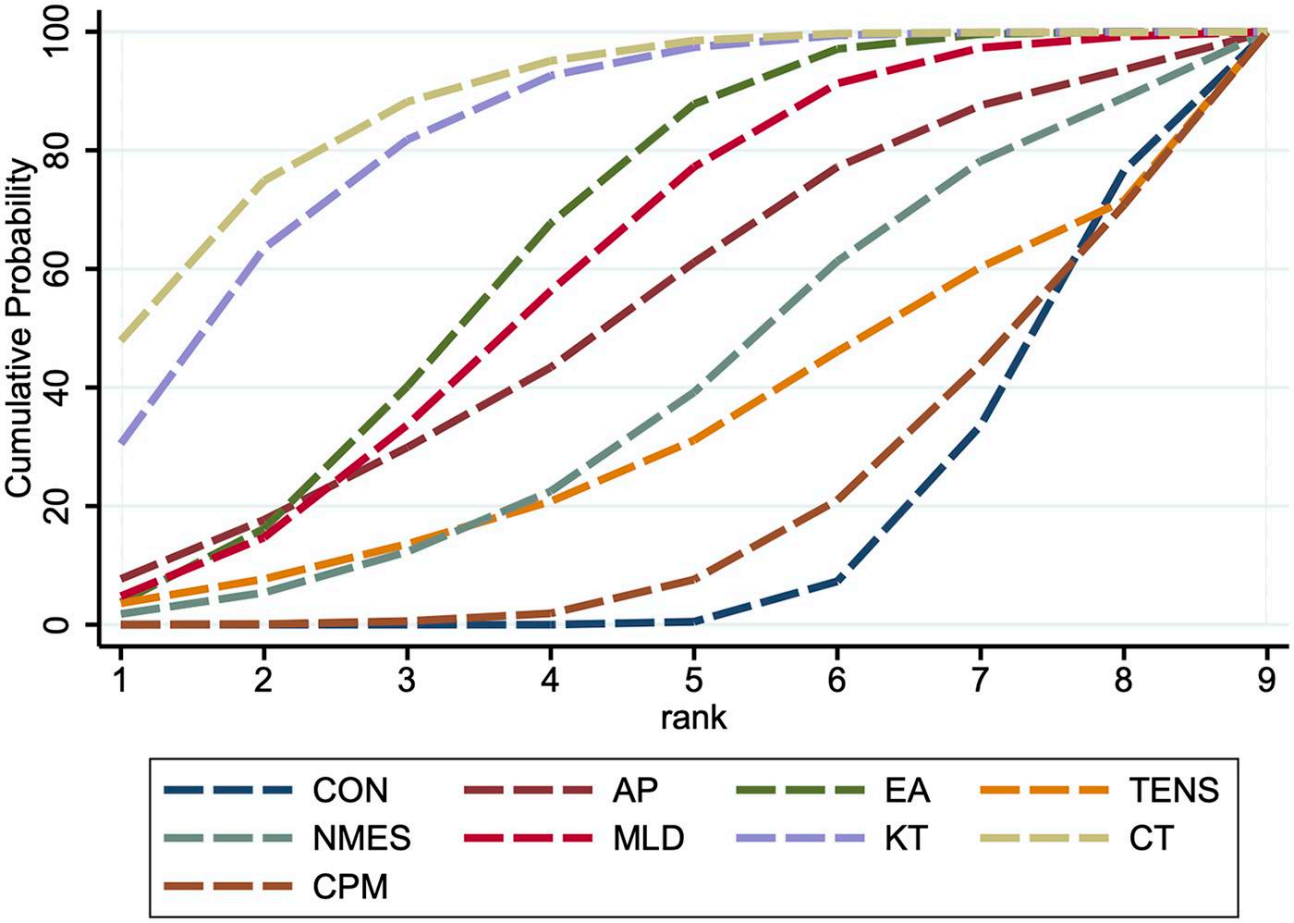
Cumulative ranking probability plots for pain. CON, control; AP, acupuncture; EA, electroacupuncture; TENS, transcutaneous electrical nerve stimulation; NMES, neuromuscular electrical stimulation; MLD, manual lymphatic drainage; KT, kinesio taping; CT, cryotherapy; CPM, continuous passive motion.

The rankings of the physical therapy modalities, based on cumulative probability plots and SUCRAs, are presented in Figure 5 and Table 1, respectively. Cryotherapy showed the highest probability of being ranked as the most effective physical therapy modality for early postoperative pain reduction following TKA, with a SUCRA value of 88.0% (Table 1) and the largest area under the curve (Figure 5), followed by kinesio taping (83.1%) and electroacupuncture (64.0%).

**Table 1:** The pain rankings for different physical therapy modalities.

Treatment	SUCRA (%)	PrBest (%)	Mean rank
CON	14.7	0.0	7.8
AP	52.3	7.7	4.8
EA	64.0	3.8	3.9
TENS	31.7	3.6	6.5
NMES	38.7	1.8	5.9
MLD	59.2	4.8	4.3
KT	83.1	30.5	2.4
CT	88.0	47.9	2.0
CPM	18.2	0.0	7.5

CON, control; AP, acupuncture; EA, electroacupuncture; TENS, transcutaneous electrical nerve stimulation; NMES, neuromuscular electrical stimulation; MLD, manual lymphatic drainage; KT, kinesio taping; CT, cryotherapy; CPM, continuous passive motion.

It should be noted that SUCRA reflects the probability of an intervention being among the most effective options within the network, rather than the magnitude of clinical effect. Therefore, SUCRA rankings should be interpreted with caution and not be considered as direct evidence of clinical superiority. In addition, uncertainty in treatment rankings should be taken into account when interpreting these results.

### 3.5 Publication bias, sensitivity analysis, and network meta-regression

Comparison-adjusted funnel plots (Supplementary Figure S5) were employed to detect potential publication bias and small-study effects. The red vertical line represents the null hypothesis, indicating that independent effect size estimates do not significantly differ from the comparison-specific pooled estimates. The visual inspection using the symmetry criterion, the funnel plot shows a roughly symmetrical distribution of RCTs on both sides, suggesting a low risk of publication bias and inappreciable small study effects.

The sensitivity analysis results are provided in Supplementary Table S8 and Supplementary Figure S6. In the NMA, sensitivity analysis did not alter the rankings or significance of interventions, indicating that our results are robust.

The results of the network meta-regression are presented in Supplementary Table S9. In brief, the variables of interest in the study had a relatively small impact on the outcomes.

## 4 Discussion

This NMA synthesized data from 42 RCTs involving 3,165 participants, providing a comprehensive comparison of various physical therapy modalities for early postoperative pain management following TKA. Regarding the risk of bias, most of the included RCTs were classified as having some concerns (*n* = 30, 71.4%), followed by low risk (*n* = 9, 21.4%) and high risk (*n* = 3, 7.2%). This pattern reflects a common methodological challenge in physical therapy research, particularly the difficulty of implementing adequate blinding procedures for both participants and personnel (78). The predominance of studies rated as “some concerns” may have important implications for the robustness of the findings, as insufficient blinding may increase the risk of performance and detection bias, potentially leading to overestimation or underestimation of treatment effects. The results suggest that electroacupuncture, MLD, kinesio taping and cryotherapy may be associated with reductions in early postoperative pain following TKA compared to the control group. Among these modalities, cryotherapy showed the highest probability of being among the more effective interventions within the network, followed by kinesio taping and electroacupuncture. Apart from electroacupuncture, kinesio taping and cryotherapy, which demonstrated statistically significant differences compared with CPM, no significant differences were observed among the remaining physical therapy modalities. However, these findings should be interpreted with caution, given the overall low to very low certainty of evidence and the potential impact of these methodological limitations.

Our study demonstrated that CPM does not significantly reduce pain following TKA compared to the control group, which supports findings from previous studies reporting limited benefits of CPM (28, 79–81). Moreover, CPM is an expensive and time-consuming procedure (76) and is often associated with increased hospital length of stay (82). Therefore, there is a need to reassess the routine use of CPM in TKA rehabilitation.

Some previous meta-analyses have controversial results for these physical therapy modalities. These studies indicated that TENS significantly reduces postoperative pain compared to the control group (30, 83), whereas MLD showed no significant advantage (29, 84). However, our results diverge, suggesting that MLD significantly reduces pain compared to the control group, while TENS does not. This discrepancy may be attributed to several factors. Firstly, the effectiveness of MLD and TENS may be influenced by stimulation parameters such as frequency, intensity, and treatment duration, which may vary across studies, leading to inconsistent results. Secondly, the baseline pain threshold and individual responses to MLD and TENS may differ among patients with TKA, influencing the treatment outcomes. Finally, it likely due to differences in inclusion criteria across studies. Although six previous RCTs examined the effects of TENS in patients with TKA, four of them do not meet our inclusion criteria. The result for TENS was based on only two studies, so a cautious interpretation is warranted. High-quality RCTs are needed to confirm its effectiveness in the future. Similarly, the limited number of studies available for certain interventions may reduce the certainty and stability of the estimated effects, and thus these findings should be interpreted cautiously.

Our study found that cryotherapy resulted in statistically significant pain reduction compared to both the control group and CPM. Cryotherapy, a low-cost and easily implementable physical therapy modality, showed the highest probability of being among the more effective interventions within the network. Its anti-inflammatory effects and ability to alleviate nociceptive pain likely contribute to its superior efficacy. Specifically, cryotherapy reduces the conduction velocity of the nerve fibers due to the asynchronous transmission in pain fibers, the release of endorphins and the inhibition of spinal neurons and an increase in the refractory period, which leads to a gradual lessening in the transmission of impulses in the sensitive nerves (85). Another mechanism suggested for the analgesic effects of cryotherapy is cold-induced vasoconstriction, which can reduce blood flow to the target tissue and reduce the release of inflammatory mediators, and also, it promotes the accumulation of local anesthetic in the target tissue (86). The combined effects of these mechanisms may explain why cryotherapy is more effective than many other treatment modalities. Despite widespread use among clinicians and the population, there is no high-quality evidence on the efficacy of cryotherapy (87). Even if mostly analgesic, cryotherapy could potentially disrupt inflammation, angiogenesis and revascularisation, delay neutrophil and macrophage infiltration as well as increase immature myofibres. This may lead to impaired tissue repair and redundant collagen synthesis (88). However, only a few studies have shown that the application of cryotherapy reduces inflammation (89–91), while most studies have shown no reduction in inflammation (92–97). As a result, the interpretation of these findings should be approached with caution, and further high-quality RCTs are needed to substantiate these results.

Similarly, kinesio taping, which modulates proprioception and reduces edema, provides a practical and non-invasive option for pain management. Kinesio taping may interact with the skin's mechanoreceptors, potentially improving proprioceptive feedback and altering the perception of pain (98, 99). This effect is linked to the activation of A-beta fibers, which modulate nociceptive signals transmitted by A-delta and C-fibers, thereby reducing pain intensity (100). Moreover, kinesio taping lifts the skin, reducing pressure on underlying tissues and promoting lymphatic fluid movement. This process helps decrease inflammation and edema, indirectly relieving pain associated with swelling (101). These mechanistic explanations warrant further exploration in future studies.

It should be noted that some physical therapy modalities, such as acupuncture (SUCRA = 52.3%) and NMES (SUCRA = 38.7%), did not demonstrate statistically significant pain reduction compared with control. Nevertheless, they ranked moderately in terms of SUCRA probabilities. This apparent discrepancy highlights an important methodological consideration: SUCRA values reflect the relative probability of an intervention being among the more effective options within the network but do not constitute direct evidence of efficacy. Therefore, readers should be cautious not to overinterpret SUCRA rankings as confirmation of clinical effectiveness in the absence of statistically significant results. Future high-quality trials with adequate sample sizes are warranted to clarify the true analgesic effects of these modalities.

### 4.1 Strengths and clinical implications

This study has several notable strengths. Firstly, this study utilized the NMA approach to conduct a comprehensive comparison of multiple physical therapy modalities on early postoperative pain following TKA, including physical therapy modalities that had not been directly compared in previous trials, addressing a critical gap in the existing literature. Secondly, we included only RCTs focusing on a single physical therapy modality (not combinations of physical therapy modalities), reducing concerns about heterogeneity between physical therapy modalities. Thirdly, the inclusion of a large sample size (3,165 participants) and diverse geographical representation (covering 14 countries) enhances the generalizability of the findings. Additionally, rigorous methodological approaches, such as the use of validated tools for risk-of-bias assessment and adherence to PRISMA-NMA guidelines, strengthen the reliability of the conclusions.

Overall, the findings of this study are of significant clinical relevance. To our knowledge, while current guidelines (102–105) consistently support the inclusion of physical therapy modalities as part of the initial clinical management for post-TKA and numerous studies (28, 30, 31, 84) have evaluated individual types of physical treatment, there is uncertainty about which treatment is best.

This study represents the first and largest NMA to compare effects of different physical therapy modalities on early postoperative pain management following TKA. The results of our study support incorporating these physical therapy modalities, into standardized postoperative care, particularly for patients aiming to reduce their reliance on pharmacological interventions. These findings underscore the potential of nonpharmacological strategies in postoperative pain management and offer a ranked comparison of their efficacy, providing an evidence-based foundation for clinical practice.

### 4.2 Study limitations and future directions

Despite its strengths, this study has certain limitations. Firstly, heterogeneity in intervention parameters across the included studies, including differences in the number of sessions, timing of initiation, duration, intensity, and co-interventions, may have influenced the results. This clinical heterogeneity is a well-recognized challenge in rehabilitation research, where variations in intervention protocols, treatment dosages, and outcome measures are often unavoidable and may substantially influence study findings (78). This complexity illustrates the inherent variability in rehabilitation interventions and outcome assessments across studies, which not only complicates the comparability of results across trials but may also challenge the transitivity assumption underlying NMA. Furthermore, the pooling of different control conditions (e.g., no intervention, placebo, and usual care) into a single node may introduce additional heterogeneity and potentially affect the validity of indirect comparisons. Although separating these control conditions in sensitivity analyses would be desirable, this was not feasible due to the limited number of studies for several comparisons, which could have resulted in disconnected networks and unstable estimates. Network meta-regression was conducted to explore the potential impact of these parameters; however, the findings should be interpreted with caution given the limited number of studies. Secondly, the follow-up periods in the included studies varied, with most focusing on short-term outcomes, which limits the discussion of the long-term effects of these physical therapy modalities. Thirdly, variability in outcome measurement tools (e.g., VAS, NRS, and the KSS pain subscale) across the included studies may have introduced measurement heterogeneity, potentially affecting the comparability of results across trials. Fourthly, an additional limitation of this study is the reliance on SMDs. Although this method was necessary to synthesize results across different pain scales (e.g., VAS, NRS, and the KSS pain subscale), SMDs are unitless and reflect relative rather than absolute changes. As a result, they are less intuitive for clinicians and patients and may reduce clinical interpretability compared with expressing outcomes on a common metric such as a 0–10 VAS. Although some statistically significant effects were observed, the clinical meaningfulness of these effect sizes remains uncertain because a validated minimal clinically important difference (MCID) threshold for postoperative pain following TKA was not applied in this meta-analysis. Therefore, the findings should be interpreted as evidence of relative comparative effects rather than definitive evidence of clinically meaningful pain reduction. Fifthly, most of the included RCTs were judged to have some concerns and did not achieve full masking, which may have led to overestimations or underestimations of the reported effects. Additionally, only studies published in English and Chinese were included, which may have introduced language bias and limited the comprehensiveness of the evidence base. Finally, the geographical distribution of the included studies is a potential limitation. Nearly half (54.8%) were conducted in China, which may affect the generalizability of our findings to Western healthcare settings. Rehabilitation practice patterns, including the intensity, frequency, and duration of therapy sessions, can differ across cultures and healthcare systems. In addition, the composition of standardized postoperative care may also vary across countries and healthcare settings. Although efforts were made to ensure that such care was applied equally within each study, differences in analgesic protocols, rehabilitation practices, and perioperative management strategies across regions may have influenced treatment effects and introduced additional clinical heterogeneity. Furthermore, patient characteristics and adherence patterns may vary between Eastern and Western populations. Although the biological mechanisms of the physical therapy modalities are likely universal, their overall effectiveness within a specific clinical pathway may be influenced by these contextual factors. Consequently, clinicians should cautiously interpret our findings within their local clinical context.

This NMA provides the following prospects and recommendations for future research. Firstly, the heterogeneity in intervention parameters across the included studies limits the comparability of results. Future RCTs should establish standardized protocols for each physical therapy modality, which would facilitate more robust comparisons. Secondly, combinations of physical therapy modalities may produce synergistic effects, hence future studies should investigate the efficacy of combining physical therapy modalities, such as cryotherapy with kinesio taping or electroacupuncture. Thirdly, the studies included in this analysis focused on early postoperative pain, typically within two weeks postoperatively. Given that TKA recovery is a long-term process, future studies should evaluate the sustained effects of physical therapy modalities on pain, functional outcomes and quality of life over extended follow-up periods. This will provide a more comprehensive understanding of their role in enhancing long-term rehabilitation. Fourthly, a substantial proportion of the included studies were judged to have some concerns regarding risk of bias. Future RCTs should improve methodological rigor, particularly in randomization and allocation concealment. Fifthly, our study pooled heterogeneous comparators (no physical therapy modality, placebo interventions, and standardized postoperative care) into a single control node. This strategy was required to preserve network connectivity and ensure adequate statistical power, given the limited number of trials available for some physical therapy modalities. Nevertheless, such pooling may compromise clinical interpretability. Future studies with a larger and more balanced evidence base should distinguish these comparators as separate nodes to improve both analytical validity and clinical applicability. Sixthly, we restricted our search to studies published in English and Chinese. Although these two languages cover the majority of high-quality RCTs in this field, relevant studies in other languages may have been missed. Future systematic reviews should consider including broader language coverage to reduce this potential bias. Additionally, to address the geographical limitation and enhance the external validity of future evidence, studies should enroll more geographically diverse populations. Specifically, future research should aim to directly compare the effectiveness of these physical therapy modalities across different healthcare models. Such head-to-head comparisons would be invaluable for clarifying whether and how cultural, systemic, or patient-level factors modulate treatment efficacy, ultimately providing more context-specific guidance for clinicians worldwide.

## 5 Conclusions

This NMA suggests that cryotherapy may be the most effective physical therapy modality for reducing early postoperative pain following TKA, followed by kinesio taping, electroacupuncture and MLD. However, these findings should be interpreted with caution due to the overall low to very low certainty of evidence and the risk of bias in the included studies. Further high-quality randomized controlled trials are needed to confirm these findings and to better inform clinical practice.

## Data Availability

The original contributions presented in the study are included in the article/Supplementary Material, further inquiries can be directed to the corresponding author/s.
