# Supplementary material for: Effects of physical therapy modalities for early postoperative pain following total knee arthroplasty: a systematic review and network meta-analysis
Source: Front Rehabil Sci. 2026 Jun 5;7:1780374. doi: 10.3389/fresc.2026.1780374 (PMC13279041; doi:10.3389/fresc.2026.1780374)
Supplement: Supplementary file 1 [file Datasheet1.pdf]

Supplementary Table S1. PRISMA 2020 checklist

Section and Topic	Item #	Checklist item	Location where item is reported
**TITLE**			
Title	1	Identify the report as a systematic review.	Title
**ABSTRACT**			
Abstract	2	See the PRISMA 2020 for Abstracts checklist.	Abstract
**INTRODUCTION**			
Rationale	3	Describe the rationale for the review in the context of existing knowledge.	Introduction
Objectives	4	Provide an explicit statement of the objective(s) or question(s) the review addresses.	Introduction
**METHODS**			
Eligibility criteria	5	Specify the inclusion and exclusion criteria for the review and how studies were grouped for the syntheses.	Methods, Study selection and eligibility criteria
Information sources	6	Specify all databases, registers, websites, organisations, reference lists and other sources searched or consulted to identify studies. Specify the date when each source was last searched or consulted.	Methods, Search strategy
Search strategy	7	Present the full search strategies for all databases, registers and websites, including any filters and limits used.	Supplementary Table S2
Selection process	8	Specify the methods used to decide whether a study met the inclusion criteria of the review, including how many reviewers screened each record and each report retrieved, whether they worked independently, and if applicable, details of automation tools used in the process.	Methods, Study selection and eligibility criteria
Data collection process	9	Specify the methods used to collect data from reports, including how many reviewers collected data from each report, whether they worked independently, any processes for obtaining or confirming data from study investigators, and if	Methods, Data extraction

Section and Topic	Item #	Checklist item	Location where item is reported
		applicable, details of automation tools used in the process.	
Data items	10a	List and define all outcomes for which data were sought. Specify whether all results that were compatible with each outcome domain in each study were sought (e.g. for all measures, time points, analyses), and if not, the methods used to decide which results to collect.	Methods, Data extraction
	10b	List and define all other variables for which data were sought (e.g. participant and intervention characteristics, funding sources). Describe any assumptions made about any missing or unclear information.	Methods, Data extraction
Study risk of bias assessment	11	Specify the methods used to assess risk of bias in the included studies, including details of the tool(s) used, how many reviewers assessed each study and whether they worked independently, and if applicable, details of automation tools used in the process.	Methods, Risk of bias and certainty assessment
Effect measures	12	Specify for each outcome the effect measure(s) (e.g. risk ratio, mean difference) used in the synthesis or presentation of results.	Methods, Outcomes
Synthesis methods	13a	Describe the processes used to decide which studies were eligible for each synthesis (e.g. tabulating the study intervention characteristics and comparing against the planned groups for each synthesis (item #5)).	Methods, Statistical analysis
	13b	Describe any methods required to prepare the data for presentation or synthesis, such as handling of missing summary statistics, or data conversions.	Methods, Statistical analysis
	13c	Describe any methods used to tabulate or visually display results of individual studies and syntheses.	Methods, Statistical analysis
	13d	Describe any methods used to synthesize results and provide a rationale for the choice(s). If meta-analysis was performed, describe the model(s), method(s) to identify the presence and extent of statistical heterogeneity, and software package(s) used.	Methods, Statistical analysis
	13e	Describe any methods used to explore possible causes of heterogeneity among study results (e.g. subgroup analysis, meta-regression).	Methods, Statistical analysis

Section and Topic	Item #	Checklist item	Location where item is reported
	13f	Describe any sensitivity analyses conducted to assess robustness of the synthesized results.	Methods, Statistical analysis
Reporting bias assessment	14	Describe any methods used to assess risk of bias due to missing results in a synthesis (arising from reporting biases).	Methods, Statistical analysis
Certainty assessment	15	Describe any methods used to assess certainty (or confidence) in the body of evidence for an outcome.	Methods, Risk of bias and certainty assessment
**RESULTS**			
Study selection	16a	Describe the results of the search and selection process, from the number of records identified in the search to the number of studies included in the review, ideally using a flow diagram.	Results, Literature selection (Figure 1)
	16b	Cite studies that might appear to meet the inclusion criteria, but which were excluded, and explain why they were excluded.	Results, Literature selection; Supplementary Table S5
Study characteristics	17	Cite each included study and present its characteristics.	Results, Studies' characteristics
Risk of bias in studies	18	Present assessments of risk of bias for each included study.	Results, Risk of bias and certainty assessment; Supplementary Figure S2
Results of	19	For all outcomes, present, for each study: (a) summary statistics for each group	Results,

Section and Topic	Item #	Checklist item	Location where item is reported
individual studies		(where appropriate) and (b) an effect estimate and its precision (e.g. confidence/credible interval), ideally using structured tables or plots.	Studies' characteristics
Results of syntheses	20a	For each synthesis, briefly summarise the characteristics and risk of bias among contributing studies.	Results, Outcomes of network meta-analysis
	20b	Present results of all statistical syntheses conducted. If meta-analysis was done, present for each the summary estimate and its precision (e.g. confidence/credible interval) and measures of statistical heterogeneity. If comparing groups, describe the direction of the effect.	Results, Outcomes of network meta-analysis
	20c	Present results of all investigations of possible causes of heterogeneity among study results.	Publication bias, sensitivity analysis, and network meta-regression
	20d	Present results of all sensitivity analyses conducted to assess the robustness of the synthesized results.	Results, Publication bias, sensitivity analysis, and network meta-regression; Supplementary Figure S6
Reporting biases	21	Present assessments of risk of bias due to missing results (arising from reporting biases) for each synthesis assessed.	Supplementary Figure S5
Certainty of evidence	22	Present assessments of certainty (or confidence) in the body of evidence for each outcome assessed.	Supplementary Table S3
**DISCUSSION**			
Discussion	23a	Provide a general interpretation of the results in the context of other evidence.	Discussion
	23b	Discuss any limitations of the evidence included in the review.	Discussion,

Section and Topic	Item #	Checklist item	Location where item is reported
			Study limitations and future directions
	23c	Discuss any limitations of the review processes used.	Discussion, Study limitations and future directions
	23d	Discuss implications of the results for practice, policy, and future research.	Discussion, Strengths and clinical implications & Study limitations and future directions.
**OTHER INFORMATION**			
Registration and protocol	24a	Provide registration information for the review, including register name and registration number, or state that the review was not registered.	Abstract
	24b	Indicate where the review protocol can be accessed, or state that a protocol was not prepared.	NA
	24c	Describe and explain any amendments to information provided at registration or in the protocol.	NA
Support	25	Describe sources of financial or non-financial support for the review, and the role of the funders or sponsors in the review.	Funding
Competing interests	26	Declare any competing interests of review authors.	Conflict of interest
Availability of data, code	27	Report which of the following are publicly available and where they can be found: template data collection forms; data extracted from included studies; data used for	Data availability

Section and Topic	Item #	Checklist item	Location where item is reported
and other materials		all analyses; analytic code; any other materials used in the review.	statement

Supplementary Table S2. Search strategy

Step	Search strategy in PubMed
#1	"Physical Therapy Modalities"[MeSH Terms] OR Physiotherap*[Title/Abstract] OR Physical Therap*[Title/Abstract] OR Neurophysiotherapy[Title/Abstract]
#2	"Transcutaneous Electric Nerve Stimulation"[MeSH Terms] OR "Transcutaneous Electric Nerve Stimulation"[Title/Abstract] OR "Transcutaneous Electric Stimulation"[Title/Abstract] OR "Percutaneous Electric Nerve Stimulation"[Title/Abstract] OR "Transcutaneous Electrical Stimulation"[Title/Abstract] OR "Transdermal Electrostimulation"[Title/Abstract] OR "Percutaneous Electrical Nerve Stimulation"[Title/Abstract] OR "Transcutaneous Electrical Nerve Stimulation"[Title/Abstract] OR "Transcutaneous Nerve Stimulation"[Title/Abstract] OR TENS[Title/Abstract] OR "Percutaneous Neuromodulation Therapy"[Title/Abstract] OR "Percutaneous Neuromodulation Therapies"[Title/Abstract] OR "Percutaneous Electrical Neuromodulation"[Title/Abstract] OR "Analgesic Cutaneous Electrostimulation"[Title/Abstract] OR Electroanalgesia[Title/Abstract]
#3	"Electric Stimulation Therapy"[MeSH Terms] OR "Electric Stimulation Therapy"[Title/Abstract] OR "Neuromuscular Electrical Stimulation"[Title/Abstract] OR "Functional Electrical Stimulation"[Title/Abstract] OR "Electrical Stimulation Therapy"[Title/Abstract] OR "Electric Stimulation Therapy"[Title/Abstract] OR NMES[Title/Abstract] OR FES[Title/Abstract] OR EMS[Title/Abstract]
#4	"Electroacupuncture"[MeSH Terms] OR Electroacupuncture[Title/Abstract]
#5	"Acupuncture"[MeSH Terms] OR Acupuncture[Title/Abstract] OR "Pharmacopuncture"[Title/Abstract]
#6	"Motion Therapy, Continuous Passive"[MeSH Terms] OR "Continuous Passive Motion"[Title/Abstract] OR "Continuous Passive Movement"[Title/Abstract] OR "CPM"[Title/Abstract]
#7	"Musculoskeletal Manipulations"[MeSH Terms] OR "Manipulation, Orthopedic "[MeSH Terms] OR "Musculoskeletal Manipulations"[Title/Abstract] OR "joint mobilization techniques"[Title/Abstract] OR "joint mobilization"[Title/Abstract] OR "manual joint mobilisation techniques"[Title/Abstract
#8	"Manual Lymphatic Drainage"[MeSH Terms] OR "Manual Lymphatic Drainage"[Title/Abstract] OR "Manual Lymph Drainage"[Title/Abstract] OR

	"Lymphatic Drainage Massage"[Title/Abstract] OR MLD[Title/Abstract]	
#9	"Athletic Tape"[MeSH Terms] OR Kinesiotape[Title/Abstract] OR Kinesiotapes[Title/Abstract] OR Kinesiotaping[Title/Abstract] OR Tape[Title/Abstract] OR Tapes[Title/Abstract] OR Taping[Title/Abstract]	
#10	"Cryotherapy"[MeSH Terms] OR Cryotherapy[Title/Abstract] OR Cryotherapies[Title/Abstract] OR "Cold Therapy"[Title/Abstract] OR "Cold Therapies"[Title/Abstract]	
#11	#1 OR #2 OR #3 OR #4 OR #5 OR #6 OR #7 OR #8 OR #9 OR #10	
#12	"Arthroplasty, Replacement, Knee"[MeSH Terms] OR "Knee Replacement Arthroplasties"[Title/Abstract] OR "Knee Replacement Arthroplasty"[Title/Abstract] OR "Total Knee Arthroplasty"[Title/Abstract] OR "Total Knee Replacement"[Title/Abstract] OR "Knee Arthroplasty"[Title/Abstract] OR "Unicompartmental Knee Arthroplasty"[Title/Abstract] OR "Unicondylar Knee Arthroplasty"[Title/Abstract] OR "Partial Knee Arthroplasty"[Title/Abstract] OR "Unicondylar Knee Replacement"[Title/Abstract] OR "Partial Knee Replacement"[Title/Abstract] OR "Unicompartmental Knee Replacement"[Title/Abstract]	
#13	"Pain"[MeSH Terms] OR Pain[Title/Abstract] OR "visual analogue scale"[Title/Abstract] OR VAS[Title/Abstract] OR "Numerical Rating Scale"[Title/Abstract] OR NRS[Title/Abstract] OR "McGill Pain Questionnaire"[Title/Abstract] OR "Western Ontario and McMaster University Osteoarthritis index"[Title/Abstract] OR WOMAC[Title/Abstract] OR "Knee Society Score"[Title/Abstract] OR KSS[Title/Abstract]	
#14	randomized controlled trial[Publication Type] OR randomized[Title/Abstract] OR placebo[Title/Abstract] OR randomised[Title/Abstract]	
#15	#11 AND #12 AND #13 AND #14	194

Step	Search strategy in EBSCOhost	
#1	AB=("Physical Therapy Modalities" OR Physiotherapy OR "Physical Therapy")	
#2	AB=("Transcutaneous Electric Nerve Stimulation" OR "Transcutaneous Electric Nerve Stimulation" OR "Transcutaneous Electric Stimulation" OR "Percutaneous Electric Nerve Stimulation" OR "Transcutaneous Electrical Stimulation" OR "Transdermal Electrostimulation" OR "Percutaneous Electrical Nerve Stimulation" OR "Transcutaneous Electrical Nerve Stimulation" OR "Transcutaneous Nerve Stimulation"	

	OR TENS OR "Percutaneous Neuromodulation Therapy" OR "Percutaneous Neuromodulation Therapies" OR "Percutaneous Electrical Neuromodulation" OR "Analgesic Cutaneous Electrostimulation" OR Electroanalgesia)	
#3	AB=("Electric Stimulation Therapy" OR "Electric Stimulation Therapy" OR "Neuromuscular Electrical Stimulation" OR "Functional Electrical Stimulation" OR "Electrical Stimulation Therapy" OR "Electric Stimulation Therapy" OR NMES OR FES OR EMS)	
#4	AB=("Electroacupuncture" OR Electroacupuncture)	
#5	AB=("Acupuncture" OR Acupuncture OR "Pharmacopuncture")	
#6	AB=("Motion Therapy, Continuous Passive" OR "Continuous Passive Motion" OR "Continuous Passive Movement" OR "CPM")	
#7	AB=("Musculoskeletal Manipulations" OR "Manipulation, Orthopedic " OR "Musculoskeletal Manipulations" OR "joint mobilization techniques" OR "joint mobilization" OR "manual joint mobilisation techniques")	
#8	AB=("Manual Lymphatic Drainage" OR "Manual Lymphatic Drainage" OR "Manual Lymph Drainage" OR "Lymphatic Drainage Massage" OR MLD)	
#9	AB=("Athletic Tape" OR Kinesiotape OR Kinesiotapes OR Kinesiotaping OR Tape OR Tapes OR Taping)	
#10	AB=("Cryotherapy" OR Cryotherapy OR Cryotherapies OR "Cold Therapy" OR "Cold Therapies")	
#11	#1 OR #2 OR #3 OR #4 OR #5 OR #6 OR #7 OR #8 OR #9 OR #10	
#12	AB=("Arthroplasty, Replacement, Knee" OR "Knee Replacement Arthroplasties" OR "Knee Replacement Arthroplasty" OR "Total Knee Arthroplasty" OR "Total Knee Replacement" OR "Knee Arthroplasty" OR "Unicompartmental Knee Arthroplasty" OR "Unicondylar Knee Arthroplasty" OR "Partial Knee Arthroplasty" OR "Unicondylar Knee Replacement" OR "Partial Knee Replacement" OR "Unicompartmental Knee Replacement")	
#13	AB=("Pain" OR Pain OR "visual analogue scale" OR VAS OR "Numerical Rating Scale" OR NRS OR "McGill Pain Questionnaire" OR "Western Ontario and McMaster University Osteoarthritis index" OR WOMAC OR "Knee Society Score" OR KSS)	
#14	AB=(randomized controlled trial OR randomized OR placebo OR randomized)	
#15	#11 AND #12 AND #13 AND #14	232

Step	Search strategy in The Cochrane Library

#1	MeSH descriptor: [Physical Therapy Modalities] explode all trees
#2	(Physical Therapy Modalities):ti,ab,kw OR (Physiotherapy):ti,ab,kw OR (Physical Therapy):ti,ab,kw
#3	#1 OR #2
#4	MeSH descriptor: [Transcutaneous Electric Nerve Stimulation] explode all trees
#5	(Transcutaneous Electrical Stimulation):ti,ab,kw OR (Transdermal Electrostimulation):ti,ab,kw OR (Percutaneous Electrical Nerve Stimulation):ti,ab,kw OR (Transcutaneous Electrical Nerve Stimulation):ti,ab,kw OR (Transcutaneous Nerve Stimulation):ti,ab,kw OR (TENS):ti,ab,kw OR (Percutaneous Neuromodulation Therapy):ti,ab,kw OR (Percutaneous Neuromodulation Therapies):ti,ab,kw OR (Percutaneous Electrical Neuromodulation):ti,ab,kw OR (Analgesic Cutaneous Electrostimulation):ti,ab,kw OR (Electroanalgesia):ti,ab,kw
#6	#4 OR #5
#7	MeSH descriptor: [Electric Stimulation Therapy] explode all trees
#8	(Electric Stimulation Therapy):ti,ab,kw OR (Neuromuscular Electrical Stimulation):ti,ab,kw OR (Functional Electrical Stimulation):ti,ab,kw OR (Electrical Stimulation Therapy):ti,ab,kw OR (Electric Stimulation Therapy):ti,ab,kw OR (NMES):ti,ab,kw OR (FES):ti,ab,kw OR (EMS):ti,ab,kw
#9	#7 OR #8
#10	MeSH descriptor: [Electroacupuncture] explode all trees
#11	(Electroacupuncture):ti,ab,kw
#12	#10 OR #11
#13	MeSH descriptor: [Acupuncture] explode all trees
#14	(Acupuncture):ti,ab,kw OR (Pharmacopuncture):ti,ab,kw
#15	#13 OR #14
#16	MeSH descriptor: [Motion Therapy, Continuous Passive] explode all trees
#17	(Continuous Passive Motion):ti,ab,kw OR (Continuous Passive Movement):ti,ab,kw OR (CPM):ti,ab,kw
#18	#16 OR #17
#19	MeSH descriptor: [Musculoskeletal Manipulations] explode all trees
#20	MeSH descriptor: [Manipulation, Orthopedic] explode all trees
#21	(joint mobilization techniques):ti,ab,kw OR (joint mobilization):ti,ab,kw OR (manual joint mobilisation techniques):ti,ab,kw

#22	19 OR #20 OR #21
#23	MeSH descriptor: [Manual Lymphatic Drainage] explode all trees
#24	(Manual Lymphatic Drainage):ti,ab,kw OR (Manual Lymph Drainage):ti,ab,kw OR (Lymphatic Drainage Massage):ti,ab,kw OR (MLD):ti,ab,kw
#25	#23 OR #24
#26	MeSH descriptor: [Athletic Tape] explode all trees
#27	(Athletic Tape):ti,ab,kw OR (Kinesiotape):ti,ab,kw OR (Kinesiotapes):ti,ab,kw OR (Kinesiotaping):ti,ab,kw OR (Tape):ti,ab,kw OR (Tapes):ti,ab,kw OR (Taping):ti,ab,kw
#28	#26 OR #27
#29	MeSH descriptor: [Cryotherapy] explode all trees
#30	(Cryotherapy):ti,ab,kw OR (Cryotherapies):ti,ab,kw OR (Cold Therapy):ti,ab,kw OR (Cold Therapies):ti,ab,kw 7,703
#31	#29 OR #30
#32	#3 OR #6 OR #9 OR #12 OR #15 OR #18 OR #22 OR #25 OR #28 OR #31
#33	MeSH descriptor: [Arthroplasty, Replacement, Knee] explode all trees
#34	(Knee Replacement Arthroplasties):ti,ab,kw OR (Knee Replacement Arthroplasty):ti,ab,kw OR (Total Knee Arthroplasty):ti,ab,kw OR (Total Knee Replacement):ti,ab,kw OR (Knee Arthroplasty):ti,ab,kw OR (Unicompartmental Knee Arthroplasty):ti,ab,kw OR (Unicondylar Knee Arthroplasty):ti,ab,kw OR (Partial Knee Arthroplasty):ti,ab,kw OR (Unicondylar Knee Replacement):ti,ab,kw OR (Partial Knee Replacement):ti,ab,kw OR (Unicompartmental Knee Replacement):ti,ab,kw
#35	#33 OR #34
#36	MeSH descriptor: [Pain] explode all trees
#37	(Pain):ti,ab,kw OR (visual analogue scale):ti,ab,kw OR (VAS):ti,ab,kw OR (Numerical Rating Scale):ti,ab,kw OR (NRS):ti,ab,kw OR (McGill Pain Questionnaire):ti,ab,kw OR (Western Ontario and McMaster University Osteoarthritis index):ti,ab,kw OR (WOMAC):ti,ab,kw OR (Knee Society Score):ti,ab,kw OR (KSS):ti,ab,kw
#38	#36 OR #37
#39	(randomized controlled trial):ti,ab,kw OR (randomized):ti,ab,kw OR (randomised):ti,ab,kw OR (placebo):ti,ab,kw OR (RCT):ti,ab,kw
#40	#32 AND #35 AND #38 AND #39 416

Step	Search strategy in Embase
#1	'arthroplasty, replacement, knee'/exp OR 'arthroplasty, replacement, knee':ab,ti OR 'knee

	replacement arthroplasties':ab,ti OR 'knee replacement arthroplasty':ab,ti OR 'total knee arthroplasty':ab,ti OR 'total knee replacement':ab,ti OR 'knee arthroplasty':ab,ti OR 'unicompartmental knee arthroplasty':ab,ti OR 'unicondylar knee arthroplasty':ab,ti OR 'partial knee arthroplasty':ab,ti OR 'unicondylar knee replacement':ab,ti OR 'partial knee replacement':ab,ti OR 'unicompartmental knee replacement':ab,ti
#2	'physical therapy modalities'/exp OR 'physiotherap*':ab,ti OR 'physical therap*':ab,ti OR 'neurophysiotherapy':ab,ti
#3	'transcutaneous electric nerve stimulation'/exp OR 'transcutaneous electric nerve stimulation':ab,ti OR 'transcutaneous electric stimulation':ab,ti OR 'percutaneous electric nerve stimulation':ab,ti OR 'transcutaneous electrical stimulation':ab,ti OR 'transdermal electrostimulation':ab,ti OR 'percutaneous electrical nerve stimulation':ab,ti OR 'transcutaneous electrical nerve stimulation':ab,ti OR 'transcutaneous nerve stimulation':ab,ti OR 'tens':ab,ti OR 'percutaneous neuromodulation therapy':ab,ti OR 'percutaneous neuromodulation therapies':ab,ti OR 'percutaneous electrical neuromodulation':ab,ti OR 'analgesic cutaneous electrostimulation':ab,ti OR 'electroanalgesia':ab,ti
#4	'electric stimulation therapy'/exp OR 'neuromuscular electrical stimulation':ab,ti OR 'functional electrical stimulation':ab,ti OR 'electrical stimulation therapy':ab,ti OR 'electric stimulation therapy':ab,ti OR 'nmes':ab,ti OR 'fes':ab,ti OR 'ems':ab,ti
#5	'electroacupuncture'/exp OR 'electroacupuncture':ab,ti
#6	'acupuncture'/exp OR 'pharmacopuncture':ab,ti
#7	'motion therapy, continuous passive'/exp OR 'continuous passive motion':ab,ti OR 'continuous passive movement':ab,ti OR 'cpm':ab,ti
#8	'musculoskeletal manipulations'/exp OR 'manipulation, orthopedic'/exp OR 'musculoskeletal manipulations':ab,ti OR 'manipulation, orthopedic':ab,ti OR 'joint mobilization techniques':ab,ti OR 'joint mobilization':ab,ti OR 'manual joint mobilisation techniques':ab,ti
#9	'manual lymphatic drainage'/exp OR 'manual lymphatic drainage':ab,ti OR 'manual lymph drainage':ab,ti OR 'lymphatic drainage massage':ab,ti OR 'mld':ab,ti
#10	'athletic tape'/exp OR 'athletic tape':ab,ti OR 'kinesiotape':ab,ti OR 'kinesiotapes':ab,ti OR 'kinesiotaping':ab,ti OR 'tape':ab,ti OR 'tapes':ab,ti OR 'taping':ab,ti
#11	'cryotherapy'/exp OR 'cryotherapy':ab,ti OR 'cryotherapies':ab,ti OR 'cold therapy':ab,ti OR 'cold therapies':ab,ti OR 'tapes':ab,ti OR 'taping':ab,ti

#12	'pain'/exp OR 'pain':ab,ti OR 'visual analogue scale':ab,ti OR 'vas':ab,ti OR 'numerical rating scale':ab,ti OR 'nrs':ab,ti OR 'mcgill pain questionnaire':ab,ti OR 'western ontario and mcmaster university osteoarthritis index':ab,ti OR 'womac':ab,ti OR 'knee society score':ab,ti OR 'kss':ab,ti
#13	'randomized controlled trial':ab,ti OR 'randomized':ab,ti OR 'randomised':ab,ti OR 'placebo':ab,ti OR 'rct':ab,ti
#14	#2 OR #3 OR #4 OR #5 OR #6 OR #7 OR #8 OR #9 OR #10 OR #11
#15	#1 AND #12 AND #13 AND #14

Step	Search strategy in Web of Science
#1	TS=("Physiotherap*" OR "Physical Therap*" OR "Neurophysiotherapy") and Preprint Citation Index (Exclude – Database)
#2	TS=(Transcutaneous Electric Nerve Stimulation OR Transcutaneous Electric Stimulation OR Percutaneous Electric Nerve Stimulation OR Transcutaneous Electrical Stimulation OR Transdermal Electrostimulation OR Percutaneous Electrical Nerve Stimulation OR Transcutaneous Electrical Nerve Stimulation OR Transcutaneous Nerve Stimulation OR TENS OR Percutaneous Neuromodulation Therapy OR Percutaneous Neuromodulation Therapies OR Percutaneous Electrical Neuromodulation OR Analgesic Cutaneous Electrostimulation OR Electroanalgesia)
#3	TS=(Electric Stimulation Therapy OR Neuromuscular Electrical Stimulation OR Functional Electrical Stimulation OR Electrical Stimulation Therapy OR Electric Stimulation Therapy OR NMES OR FES OR EMS)
#4	TS=(Electroacupuncture)
#5	TS=(Acupuncture OR Pharmacopuncture)
#6	TS=(Continuous Passive Motion OR Continuous Passive Movement OR CPM)
#7	TS=(Musculoskeletal Manipulations OR Manipulation, Orthopedic OR joint mobilization techniques OR joint mobilization OR manual joint mobilisation techniques)
#8	TS=(Manual Lymphatic Drainage OR Manual Lymph Drainage OR Lymphatic Drainage Massage OR MLD)
#9	TS=(Athletic Tape OR Kinesiotape OR Kinesiotapes OR Kinesiotaping OR Tape OR Tapes OR Taping)
#10	TS=(Cryotherapy OR Cryotherapies OR Cold Therapy OR Cold Therapies)
#11	#10 OR #9 OR #8 OR #7 OR #6 OR #5 OR #4 OR #3 OR #2 OR #1
#12	TS=(Knee Replacement Arthroplasties OR Knee Replacement Arthroplasty OR Total

	Knee Arthroplasty OR Total Knee Replacement OR Knee Arthroplasty OR Unicompartmental Knee Arthroplasty OR Unicondylar Knee Arthroplasty OR Partial Knee Arthroplasty OR Unicondylar Knee Replacement OR Partial Knee Replacement OR Unicompartmental Knee Replacement)	
#13	TS=(Pain OR visual analogue scale OR VAS OR Numerical Rating Scale OR NRS OR McGill Pain Questionnaire OR Western Ontario and McMaster University Osteoarthritis index OR WOMAC OR Knee Society Score OR KSS)	
#14	TS=(randomized controlled trial OR randomized OR randomised OR placebo OR RCT)	
#15	#11 AND #12 AND #13 AND #14	500

Step	Search strategy in CNKI	
#1	（主题：全膝关节置换）	
#2	（主题：物理治疗 + 物理疗法 + 针灸 + 电针+ 经皮神经电刺激 + 神经肌肉电刺激 + 徒手淋巴引流 + 冰敷+ 冷疗 + 持续被动运动 + 持续被动活动 + 关节松动）	
#3	（主题：疼痛）	
#4	（主题：随机对照）	
#5	#1 AND #2 AND #3 AND #4	203

Note: SPORTDiscus and CINAHL Plus are all accessible through EBSCOhost for searching.

Supplementary Table S3. The CINeMA for NMA

Comparison	N	Within-study bias	Reporting bias	Indirectness	Imprecision	Heterogeneity	Incoherence	Confidence rating
Mixed evidence								
CON vs AP	3	Some concerns	Some concerns	No concerns	Some concerns	Some concerns	No concerns	Very low
CON vs EA	10	Some concerns	No concerns	No concerns	No concerns	Some concerns	No concerns	Low
CON vs TENS	2	Some concerns	Some concerns	No concerns	Some concerns	Some concerns	No concerns	Very low
CON vs NMES	4	Some concerns	Some concerns	No concerns	Some concerns	Some concerns	No concerns	Very low
CON vs MLD	4	No concerns	Some concerns	No concerns	No concerns	Some concerns	No concerns	Low
CON vs KT	5	Some concerns	Some concerns	No concerns	No concerns	Some concerns	No concerns	Very low
CON vs CT	5	Some concerns	Some concerns	No concerns	No concerns	Some concerns	No concerns	Very low
CON vs CPM	8	Some concerns	Some concerns	No concerns	No concerns	Major concerns	No concerns	Very low
EA vs MLD	1	Some concerns	Some concerns	No concerns	No concerns	Major concerns	No concerns	Very low
MLD vs KT	2	Some concerns	Some concerns	No concerns	Some concerns	Some concerns	No concerns	Very low
KT vs CT	1	Some concerns	Some concerns	No concerns	No concerns	Major concerns	No concerns	Very low
CT vs CPM	1	Some concerns	Some concerns	No concerns	No concerns	Some concerns	No concerns	Very low
Indirect evidence								
AP vs EA		Some concerns	Some concerns	No concerns	Some concerns	Some concerns	No concerns	Very low
AP vs TENS		Some concerns	Some concerns	No concerns	Some concerns	Some concerns	No concerns	Very low
AP vs NMES		Some concerns	Some concerns	No concerns	Some concerns	Some concerns	No concerns	Very low
AP vs MLD		Some concerns	Some concerns	No concerns	Some concerns	Some concerns	No concerns	Very low
AP vs KT		Some concerns	Some concerns	No concerns	Some concerns	Some concerns	No concerns	Very low
AP vs CT		Some concerns	Some concerns	No concerns	Some concerns	Some concerns	No concerns	Very low
AP vs CPM		Some concerns	Some concerns	No concerns	Some concerns	Some concerns	No concerns	Very low
EA vs TENS		Some concerns	Some concerns	No concerns	Some concerns	Some concerns	No concerns	Very low
EA vs NMES		Some concerns	Some concerns	No concerns	Some concerns	Some concerns	No concerns	Very low
EA vs KT		Some concerns	Some concerns	No concerns	Some concerns	Some concerns	No concerns	Very low
EA vs CT		Some concerns	Some concerns	No concerns	Some concerns	Some concerns	No concerns	Very low
EA vs CPM		Some concerns	Some concerns	No concerns	No concerns	Some concerns	No concerns	Very low
TENS vs NMES		Some concerns	Some concerns	No concerns	Major concerns	No concerns	No concerns	Very low
TENS vs MLD		No concerns	Some concerns	No concerns	Some concerns	Some concerns	No concerns	Very low
TENS vs KT		Some concerns	Some concerns	No concerns	Some concerns	No concerns	No concerns	Very low
TENS vs CT		Some concerns	Some concerns	No concerns	Some concerns	No concerns	No concerns	Very low
TENS vs CPM		Some concerns	Some concerns	No concerns	Major concerns	No concerns	No concerns	Very low
NMES vs MLD		Some concerns	Some concerns	No concerns	Some concerns	Some concerns	No concerns	Very low
NMES vs KT		Some concerns	Some concerns	No concerns	Some concerns	Some concerns	No concerns	Very low
NMES vs CT		Some concerns	Some concerns	No concerns	Some concerns	No concerns	No concerns	Very low
NMES vs CPM		Some concerns	Some concerns	No concerns	Some concerns	Some concerns	No concerns	Very low
MLD vs CT		Some concerns	Some concerns	No concerns	Some concerns	Some concerns	No concerns	Very low
MLD vs CPM		Some concerns	Some concerns	No concerns	Some concerns	No concerns	No concerns	Very low
KT vs CPM		Some concerns	Some concerns	No concerns	No concerns	Some concerns	No concerns	Very low

Note: CON: control; AP: acupuncture; EA: electroacupuncture; TENS: transcutaneous electrical nerve stimulation; NMES: neuromuscular electrical stimulation; MLD: manual lymphatic drainage; KT: kinesio taping; CT: cryotherapy; CPM: continuous passive motion.

To assess the credibility of each comparison, we used CINeMA[1], with reference to practices in previous literature[2,3]. Similar to GRADE, we considered the evidence for comparisons to show high confidence then downgraded on the basis of concerns in each domain, as follows:

Within study bias: Comparisons were downgraded when most of the studies providing direct evidence for comparisons were unclear or high risk.

Reporting bias[4]: For each comparison with at least 10 studies, we created funnel plots, and conducted a Egger's regression test. Given these tests are not recommended for comparisons with fewer than 10 studies, those comparisons were considered to show "some concerns."

Indirectness: Our primary population of interest was adults following total knee arthroplasty. Studies were considered to be indirect if they focused on one sex only (>90% male or female), participants with comorbidities (eg, heart disease) or young

adults (18-35 years). We flagged these studies as showing some concerns if one of these factors was present, and as “major concerns” if two of these factors were present.

Imprecision: As per CINeMA, we used the clinically important difference of Hedges’ g=0.8 to ascribe a zone of equivalence, where differences were not considered clinically significant ($-0.8<g<0.8$). Studies were flagged as some concerns for imprecision if the bounds of the 95% credible interval extended across that zone, and they were flagged as major concerns if the bounds extended to the other side of the zone of equivalence (such that effects could be harmful).

Heterogeneity: Prediction intervals account for heterogeneity differently from credible intervals. As a result, CINeMA accounts for heterogeneity by assessing whether the prediction intervals and the credible intervals lead to different conclusions about clinical significance (using the same zone of equivalence from imprecision). Comparisons are flagged as some concerns if the prediction interval crosses into, or out of, the zone of equivalence once (eg, from helpful to no meaningful effect), and as major concerns if the prediction interval crosses the zone twice (eg, from helpful and harmful).

Incoherence: Incoherence assesses whether the network meta-analysis provides similar estimates when using direct evidence compared with indirect evidence. Incoherence provides some evidence the network may violate the assumption of transitivity: that the only systematic difference between arms is the treatment, not other confounders. The content regarding inconsistency tests has been described in the statistical analysis section of the main text. We flagged comparisons as some concerns if either no direct comparisons were available or direct and indirect evidence gave different conclusions about clinical significance (eg, from helpful to no meaningful effect, as per imprecision and heterogeneity). Again, we classified comparisons as major concerns if the direct and indirect evidence changed the sign of the effect or changed both limitsof the credible interval.

[1]Papakonstantinou T, Nikolakopoulou A, Higgins JPT, Egger M, Salanti G. CINeMA: Software for semiautomated assessment of the confidence in the results of network meta-analysis. Campbell Syst Rev. 2020;16(1):e1080. Published 2020 Mar 11. doi:10.1002/cl2.1080

[2]Noetel M, Sanders T, Gallardo-Gómez D, et al. Effect of exercise for depression: systematic review and network meta-analysis of randomised controlled trials. BMJ. 2024;384:e075847. Published 2024 Feb 14. doi:10.1136/bmj-2023-075847

[3]De Crescenzo F, D'Alò GL, Ostinelli EG, et al. Comparative effects of pharmacological interventions for the acute and long-term management of insomnia disorder in adults: a systematic review and network meta-analysis. Lancet. 2022;400(10347):170-184. doi:10.1016/S0140-6736(22)00878-9

[4]Sterne JAC, Sutton AJ, Ioannidis JPA, et al. Recommendations for examining and interpreting funnel plot asymmetry in meta-analyses of randomised controlled trials. BMJ 2011;343:d4002. 10.1136/bmj.d4002

Supplementary Table S4. The STATA code for NMA

network setup mean sd n, studyvar(study) trtvar(trt) smd

#set up data from arm-specific counts

network convert pair (augment)

#convert data between formats

networkplot treat1 treat2, lab("treatment name 1" "treatment name 2" "treatment name 3"...) title("***") nodec(navy) graphregion(fcolor(white))

#draw the plot of a network in terms of nodes and edges

ifplot SMD seSMD treat1 treat2 study, tau2(loop) lab("treatment name 1" "treatment name 2" "treatment name 3"...)

#evaluation of statistical inconsistency in networks of interventions.

netfunnel SMD seSMD treat1 treat2, bycomparison add(lfit _stderr _ES_CEN)

draw the comparison-adjusted funnel plot

network meta i (c)

network meta defines a model to be fit: either the consistency model or the design-by-treatment interaction inconsistency model

network meta c, **regress(varlist)**

#specifies covariates for network meta-regression. Every treatment contrast is allowed to depend on the covariate(s) listed. This option is currently only allowed in the augmented format.

network sidesplit all, tau

#fit side-splitting (node-splitting) model(s)

netleague

#league table for networks of interventions.

intervalplot, pred null(0) lab(Placebo ARNI ARB ACEI BB D CBB) notab 连续

#draw a forest plot with the network estimates and their confidence and predictive intervals

network rank min, bar zero all reps(10000) gen(prob)

#rank treatments after network meta-analysis

Note: This section provides sample STATA code used in the literature, with brief explanations following the # symbol. For specific details, please refer to the STATA help documentation.

Supplementary Table S5. Description of Studies Excluded at Full Text Stage

Author	Title	Reason for exlcusion
Zhang 2024	Effectiveness and safety of auricular acupuncture on adjuvant analgesia in patients with total knee arthroplasty: a randomized sham-controlled trial	Lack of extractable outcomes
Yuksel 2016	Comparison between kinesiotaping and cold therapy on muscle strength functional performance outcomes after total knee arthroplasty: Preliminary results of a randomized controlled trial	Not RCT
Yueyan 2020	Study on functional recovery of acupuncture after knee arthroplasty	Not RCT
Yang 2013	Compare the Traditional Chinese Medicine manipulation with rehabilitation on in-patients after total knee arthroplasty	Lack of proper control
Xu 2020	Using electroacupuncture to recover muscle strength in patients with knee osteoarthritis after total knee arthroplasty: a study protocol for a double-blinded, randomized, and placebo-controlled trial	Not RCT
Wittig-Wells 2015	Does the use of a brief cryotherapy intervention with analgesic administration improve pain management after total knee arthroplasty?	Lack of proper control
Wirries 2020	Impact of continuous passive motion on rehabilitation following total knee arthroplasty	Lack of extractable outcomes
Webb 1998	The use of cold compression dressings after total knee replacement: a randomized controlled trial	Ineligible participants
Walker 1991	Postoperative use of continuous passive motion, transcutaneous electrical nerve stimulation, and continuous cooling pad following total knee arthroplasty	Lack of proper control
VergiLi 2022	The effect of manual lymphatic drainage on postoperative recovery process following total knee arthroplasty	Lack of proper control
Tzeng 2015	Single-Blinded, Randomised Preliminary Study Evaluating the Effects of 2 hz Electroacupuncture for Postoperative Pain in Patients with Total Knee Arthroplasty	Lack of extractable outcome
Toth 2016	Skeletal Muscle Atrophy and Dysfunction Following Total Knee Arthroplasty	Not RCT
Thijs 2019	Reduced opiate use after total knee arthroplasty using computer-assisted cryotherapy	Lack of proper control
Stevens-Lapsley	Early neuromuscular electrical stimulation to improve quadriceps muscle	Lack of extractable

2012	strength after total knee arthroplasty: a randomized controlled trial	outcomes
Sobiech 2022	Does Application of Lymphatic Drainage with Kinesiology Taping Have Any Effect on the Extent of Edema and Range of Motion in Early Postoperative Recovery following Primary Endoprosthetics of the Knee Joint?	Lack of extractable outcomes
Sahni 2022	Effectiveness of continuous passive motion and conventional physical therapy after total knee arthroplasty: a randomized clinical trial	Lack of extractable outcomes
Rubin 2023	Acupuncture Effect on Analgesic Consumption and Cortisol Levels Following Total Knee Arthroplasty: A Randomized Controlled Trial	Lack of extractable outcomes
Richter 2022	Effect of continuous passive motion on the early recovery outcomes after total knee arthroplasty	Lack of extractable outcomes
Rakel 2014	Transcutaneous electrical nerve stimulation for the control of pain during rehabilitation after total knee arthroplasty: A randomized, blinded, placebo-controlled trial	Lack of extractable outcomes
Radkowski 2007	Cryotherapy temperature differences after total knee arthroplasty: a prospective randomized trial	Lack of proper control
Pope 1997	Continuous passive motion after primary total knee arthroplasty. Does it offer any benefits?	Lack of extractable outcomes
Pichonnaz 2013	Outcome of manual lymphatic drainage on swelling, range of motion and pain following total knee arthroplasty	Not RCT
Pichonnaz 2015	Effects of manual lymphatic drainage following total knee arthroplasty: A prospective randomised controlled trial	Not RCT
Petersen 2018	No effect of Acupuncture as adjunctive therapy for patients with total knee replacement: A randomized controlled trial	Lack of extractable outcomes
Pereira 2014	The effects of end-of-range grade A+ mobilisation following acute primary TKA	Not RCT
Pereira 2015	The effect of end-of-range grade A+ knee mobilisation following acute primary total knee arthroplasty: a randomised controlled trial	Inappropriate intervention
Park 2021	Efficacy and safety of acupuncture treatment as an adjunctive therapy after knee replacement: Single-center, pragmatic, randomized, assessor blinded, pilot study	Not RCT
Oktas 2018	The effect of intensive exercise program and kinesiotaping following total knee arthroplasty on functional recovery of patients	Lack of extractable outcomes

Noh 2015	Changes in pain, swelling, and range of motion according to physical therapy intervention after total knee arthroplasty in elderly patients	Lack of proper control
Nishigami 2019	A pleasant sensation evoked by knee or hand icing influences the effect on pain intensity in patients after total knee arthroplasty: A prospective, randomized, cross-over study	Lack of proper control
Morsi 2002	Continuous-flow cold therapy after total knee arthroplasty	Lack of proper control
McInnes 1992	A controlled evaluation of continuous passive motion in patients undergoing total knee arthroplasty	Ineligible participants
Mayoral 2013	Efficacy of myofascial trigger point dry needling in the prevention of pain after total knee arthroplasty: A randomized, double-blinded, placebo-controlled trial	Inappropriate intervention
Maniar 2012	To use or not to use continuous passive motion post-total knee arthroplasty presenting functional assessment results in early recovery	Ineligible participants
MacDonald 2000	Prospective randomized clinical trial of continuous passive motion after total knee arthroplasty	Lack of extractable outcomes
Ma 2021	Effect of crossing acupoints of the same name of hands and feet on pain after total knee arthroplasty	Lack of extractable outcomes
Licciardone 2004	A randomized controlled trial of osteopathic manipulative treatment following knee or hip arthroplasty	Ineligible participants
Li 2019	Efficacy of different-frequency TEAS on acute pain after the total knee arthroplasty: a study protocol for a parallel group randomized trial	Not RCT
Levy 1993	The role of cold compression dressings in the postoperative treatment of total knee arthroplasty	Lack of proper control
Lenssen 2008	Effectiveness of prolonged use of continuous passive motion (CPM), as an adjunct to physiotherapy, after total knee arthroplasty	Lack of proper control
Lenssen 2006	Effectiveness of prolonged use of continuous passive motion (CPM) as an adjunct to physiotherapy following total knee arthroplasty: design of a randomised controlled trial	Not RCT
Leach 2006	Continuous passive motion following total knee replacement: a prospective randomized trial with follow-up to 1 year	Lack of extractable outcomes
Kuyucu 2015	Is cold therapy really efficient after knee arthroplasty?	Ineligible participants
Kim 2009	Clinical value of regular passive ROM exercise by a physical therapist after total knee arthroplasty	Inappropriate intervention

Ivey 1994	Cryotherapy for postoperative pain relief following knee arthroplasty	Lack of proper control
Huffman 2024	Nurse initiated auricular acupressure for post-operative pain control in patients undergoing TKA or THA: A randomized controlled trial	Ineligible participants
Huang 2003	The effect of continuous passive motion after total knee arthroplasty on joint function	Lack of extractable outcomes
Huang 2022	Low-level laser acupuncture reduces postoperative pain and morphine consumption in older patients with total knee arthroplasty: A randomized placebo-controlled trial	Lack of extractable outcomes
Herbold 2014	Randomized Controlled Trial of the Effectiveness of Continuous Passive Motion After Total Knee Replacement	Lack of extractable outcomes
He 2013	Auricular acupressure for analgesia in perioperative period of total knee arthroplasty	Inappropriate intervention
Hasubhai 2017	Effectiveness of Conventional Physiotherapy along with Continuous Passive Motion after Total Knee Arthroplasty	Lack of extractable outcomes
Harms 1991	Continuous passive motion as an adjunct to treatment in the physiotherapy management of the total knee arthroplasty patient	Ineligible participants
Guney Deniz 2018	Comparison of kinesio tape application and manual lymphatic drainage on lower extremity oedema and functions after total knee arthroplasty	Not RCT
Gibbons 2001	Cryotherapy compared with Robert Jones bandage after total knee replacement: a prospective randomized trial	Lack of proper control
Gao 2011	Comprehensive rehabilitation following total knee arthroplasty: a randomized controlled trial	Lack of proper control
Fujiura 2020	Effect of manual lymph drainage for up to 10 days after total knee arthroplasty: Arandomized controlled trial	Lack of extractable outcomes
Eto 2023	Analgesic Effect of Passive Range-of-Motion Exercise on the Healthy Side for Pain after Total Knee Arthroplasty: A Prospective Randomized Trial	Lack of proper control
Drks 2016	Effect of Mulligan´s MWM-Technique to improve knee flexion in total knee replacement patients in the rehabilitation phase - A randomized controlled trial	Not RCT
Donec 2014	The effectiveness of Kinesio Taping® after total knee replacement in early postoperative rehabilitation period. A randomized controlled trial	Inappropriate intervention
Desteli 2015	Effect of both preoperative and postoperative cryoceutical treatment on	Lack of extractable

	hemostasis and postoperative pain following total knee arthroplasty	outcomes
Denis 2006	Effectiveness of continuous passive motion and conventional physical therapy after total knee arthroplasty: a randomized clinical trial	Lack of proper control
Demircioglu 2015	The effect of neuromuscular electrical stimulation on functional status and quality of life after knee arthroplasty: a randomized controlled study	Inappropriate intervention
Colwell 1992	The influence of continuous passive motion on the results of total knee arthroplasty	Lack of extractable outcomes
CiHan 2021	Outcomes with Additional Manual Lymphatic Drainage to Rehabilitation Protocol in Primary Total Knee Arthroplasty Patients: Preliminary Clinical Results	Lack of proper control
Chang 2012	Auricular acupressure for managing postoperative pain and knee motion in patients with total knee replacement: A randomized sham control study	Inappropriate intervention
Cao 2023	Effect of Wrist-Ankle Acupuncture on Postoperative Analgesia after Total Knee Arthroplasty	Ineligible participants
Brouwers 2022	The role of computer-assisted cryotherapy in the postoperative treatment after total knee arthroplasty: positive effects on pain and opioid consumption	Lack of extractable outcomes
Breit 2004	Transcutaneous electrical nerve stimulation for postoperative pain relief after total knee arthroplasty	Lack of extractable outcomes
Boggs 2016	Electrical stimulation to reduce pain and improve function after knee replacement	Not RCT
Boese 2014	The efficacy of continuous passive motion after total knee arthroplasty: a comparison of three protocols	Ineligible participants
Bennett 2005	A Comparison of 2 Continuous Passive Motion Protocols After Total Knee Arthroplasty	Lack of extractable outcomes
Beaupré 2001	Exercise combined with continuous passive motion or slider board therapy compared with exercise only: A randomized controlled trial of patients following total knee arthroplasty	Ineligible participants
Avramidis 2011	Does electric stimulation of the vastus medialis muscle influence rehabilitation after total knee replacement?	Lack of extractable outcomes
Angulo 1990	Use of Postoperative TENS and Continuous Passive Motion Following Total Knee Replacement	Lack of proper control
Alkire 2010	Use of inpatient continuous passive motion versus no CPM in computer-	Ineligible participants

	assisted total knee arthroplasty	
Alaca 2015	Comparison of the long-term effectiveness of progressive neuromuscular facilitation and continuous passive motion therapies after total knee arthroplasty	Lack of proper control
Adan 2021	Adjunctive Acupuncture within a Total Joint Replacement Program	Not RCT
Actrn 2020	The effect of kinesiotaping on the temperature distribution and intensity of pain at the site of application. A randomized clinical trial	Not RCT
Zheng 2011	Effect of neuromuscular electrical stimulation of the quadriceps muscles on functional rehabilitation after total knee arthroplasty	Lack of extractable outcomes
Xing 2017	Observation on the efficacy of rehabilitation exercise in improving joint function after total knee arthroplasty	Lack of proper control
Shao 2023	The effect of TCM acupuncture therapy on the early rehabilitation of patients after total knee arthroplasty	Not RCT
Liu 2015	Effect of intervention on sustained passive movement after total knee arthroplasty	Inappropriate intervention
Kang 2022	Efficacy of electroacupuncture combined with celecoxib in the treatment of pain after total knee arthroplasty	Lack of extractable outcomes
Guan 2018	Comparison of the effect of CPM exercise after knee arthroplasty with exercise under the guidance of a rehabilitation therapist	Inappropriate intervention

Supplementary Table S6. Study characteristics

Study	Country	Intervention	No. of patients	Age,Mean (SD/Range)	Gender (n,% female)	Pain Scale	Baseline Pain （Mean±SD）
Chen 2019	China	EA: 7 times a week for 30 minutes for 1 week	EA: 20	EA: 67 (7)	EA: 18（90）	VAS	5.03±1.3
		CON	CON: 20	CON: 67 (6)	CON: 20 (100)		5.31±1.26
Chen 2015b	China	EA: once a day for 30min for 1 week	EA: 20	EA: 65.6（52-79）	EA: 10（50）	VAS	5.13±1.97
		MLD: twice a day for 30min for 1 week	MLD: 20	MLD: 62（55-81）	MLD: 12（60）		4.13±2.38
Huang 2023	China	EA: once a day for 30min for 3 days	EA: 38	EA: 68.8 (5.4)	EA: 36 (94.7)	VAS	31.92±9.74
		CON	CON: 38	CON: 67.6 (4.9)	CON: 31 (81.6)		31.61±8.56
Kang 2023	China	EA: once day for 20min for 5 days	EA: 16	EA: 71.4 (6.1)	EA: 14（87.5）	NRS	6±0.6
		CON	CON: 15	CON: 71.4 (6.1)	CON: 13 (86.7)		6.1±0.6
Mao 2021	China	EA: once a day for 30min for 2 days	EA: 45	EA: 62.4 (7.1)	EA: 19（42.2）	VAS	6.13±1.27
		CON	CON: 45	CON: 63.3 (7.8)	CON: 17 (37.8)		6.62±1.13
Sun 2018	China	EA: once a day for 20min for 2 weeks	EA: 30	NR	NR	VAS	6.03±1.07
		CON	CON: 30	NR	NR		6.67±1.03
Shi 2022	China	EA: once a day for 30min for 3 days	EA: 40	EA: 66.7 (4.6)	EA: 22(55)	VAS	5.8±0.9
		CON	CON: 40	CON: 67 (4.9)	CON:24(60)		5.9±0.9
Xiang 2024	China	EA: once a day for 30min for 1 week	EA: 41	EA: 63 (5)	EA: 18 (43.9)	VAS	2.13±0.64
		CON	CON: 41	CON: 63 (6)	CON: 16(39.1)		2.48±0.71
Yang 2017	China	EA: twice a day for 30min for 1 week	EA: 30	EA: 67.3(59.6-78.4)	EA: 24(80)	VAS	4.17±0.83
		CON	CON: 30	CON: 68.5(58.4-80.3)	CON:23(76.7)		3.77±0.89

Zhang 2020	China	EA: once a day for 30min for 2 weeks	EA: 49	EA: 68 (4. 8)	EA: 30 (61.22)	VAS	3.06±0.95
		CON	CON: 49	CON: 69 (5. 3)	CON: 27 (55.1)		3.21±1.04
Zhang 2023a	China	EA: once a day for 30min for 2 weeks	EA: 47	EA: 62.6 (5. 3)	EA: 21 (44.7)	NRS	6.54±1.58
		CON	CON: 47	CON: 63.2 (5.9)	CON: 18 (44.7)		6.71±1.63
Chen 2015a	China	AC: once for 20 minutes	AC: 30	AC: 68.9 (9)	AC: 27 (90)	VAS	60±16
		CON	CON: 30	CON: 69.0 (8.6)	CON: 17 (60.7)		57±18
Mikashima 2012	Japan	AC: 3 times a week for 20 minutes for 1 week	AC: 40	AC: 72 (7)	AC: 30 (75)	VAS	60±6
		CON	CON: 40	CON: 73 (5)	CON: 28 (70)		59±5
Tsang 2007	China	AC：once for 20 minutes	AC: 15	AC: 70.6(5.8)	AC: 12（80）	NRS	2.7±2
		CON	CON: 15	CON: 66.1 (7.5)	CON: 12（80）		3.1±2.4
Beckwée 2018	Belgium	TENS: once a day for 40min for 5 days	TENS: 25	TENS: 71.8 (7.3)	TENS: 17 (68.0)	VAS	32.4±30.7
		CON	CON: 28	CON: 72.9 (7.6)	CON: 17 (60.7)		17.3±24.7
Zhang 2014	China	TENS: once a day for 2 weeks	TENS: 30	TENS: 70.3 (6.5)	TENS: 21 (70)	VAS	4.08±1.46
		CON	CON: 30	CON: 66.9 (8.7)	CON: 19 (63.3)		3.99±1.28
Dabadghav 2019	India	NMES: once a day for 20min for 1 week	NMES: 28	NMES: 60.8 (5.7)	NMES: 16 (57)	VAS	6.79±1.23
		CON	CON: 28	CON: 60.2 (5.7)	CON: 16 (57)		6.29±1.36
Levine 2013	America	NMES: once a day for 6 weeks	NMES: 35	NMES: 68.1	NMES: 25 (76)	KSS	49.6±13.9
		CON	CON: 35	CON: 65.1	CON: 21 (62)		48.1±12.5
Stevens-Lapsley	America	NMES: twice a day for 6 weeks	NMES: 35	NMES: 66.2 (9.1)	NMES: 20 (57.1)	VAS	2.5±1.9
2008		CON	CON: 31	CON: 64.8 (7.7)	CON: 16 (51.6)		2.7±2.5
Yoshida 2017	Japan	NMES: 5 times a week for 30min for 2 weeks	NMES: 22	NMES: 75.9 (4.7)	NMES: 18 (81.8)	VAS	53±16
		CON	CON: 22	CON: 72.5 (6.2)	CON: 22 (90.9)		53±27
Ebert 2013	Australia	MLD: once a day for 30min for 3 days	MLD: 24	MLD: 70.8 (48-89)	MLD: 7(29)	NRS	4.67±1.59

		CON	CON: 26	CON: 69.2 (51-87)	CON: 7(27)		4.71±1.64
Feng 2023	China	MLD: once a day for 30-40 min for 1 week	MLD: 34	NR	NR	VAS	3.47±1.55
		CON	CON: 34				2.86±0.92
Pichonnaz 2016	Switzerland	MLD: once a day for 30 min for 5 days	MLD: 30	MLD: 71.3 (8.5)	MLD: 62.1	VAS	25.9±21.5
		CON	CON: 30	CON: 70.1 (9.1)	CON: 67.7		31±28.4
Guney-Deniz 2023	Turkey	MLD: once a day for 4 days	MLD: 13	MLD: 65.5(3.3)	MLD: 13 (100)	VAS	6.5±1.2
		KT: 24h a day for 4 days	KT: 12	KT: 65.4(3.9)	KT: 12 (100)		5.9±2.3
Tornatore 2020	Italy	MLD: once a day for 2 days	MLD: 33	MLD: 71.3 (7.1)	MLD: 26 (78.8)	NRS	7.27±1.44
		KT: 24h a dayfor 4 days	KT: 33	KT: 72.8 (7.3)	KT: 25 (5.6)		7.55±1.28
Cakmak 2023	Turkey	KT: 24h a day for 1 week	KT: 62	KT: 66.1 (6.2)	KT: 56 (90.3)	VAS	9±1.08
		CON	CON: 63	CON: 64 (8.5)	CON: 55 (87.3)		9.02±0.95
Wang 2023	China	KT: 24h a day for 28 days	KT: 46	KT: 47.3 (8.2)	KT: 21 (45.6)	VAS	4.72±0.55
		CON	CON: 46	CON: 48.2 (8.1)	CON: 23 (50)		4.64±0.59
Xue 2021	China	KT: 24h a day for 30 days	KT: 63	NR	NR	VAS	4.52±0.57
		CON	CON: 64				4.76±0.79
Yuksel 2022	Turkey	KT: 24h a day for 6 days	KT: 33	KT: 65.4 (6.7)	KT: 25 (75.6)	NRS	7.62±1.52
		CT: 12-15min every 2 h for 6 days	CT: 34	CT: 66.8 (9.8)	CT: 23 (69.7)		6.86±2.18
Kullenberg 2006	Sweden	CT: once every 1 h for 3 days (mean 60 h)	CT: 43	CT: 68.1 (6)	CT: 25 (58)	VAS	2.1±1
		CON	CON: 40	CON: 68.9 (6.8)	CON: 24 (60)		2.2±0.8
Li 2023	China	CT: once every 4 h for 30min for 2 days	CT: 38	CT: 57.1 (5.7)	CT: 23 (60.5)	VAS	6.25±0.63
		CON	CON: 38	CON: 56.7 (5.7)	CON: 24 (63.2)		6.23±0.61
Li 2021	China	CT: 2-3h a day for 3 days	CT: 55	CT: 72.3 (4.3)	CT: 30 (54.5)	VAS	6.33±1.25
		CON	CON: 55	CON: 73.4 (4.6)	CON: 33 (60)		6.45±1.28
Pan 2014	China	CT: 3 times a day for 20 min for 2 days	CT: 68	CT: 64.1	CT: 40 (58.9)	VAS	5.14±1.02
		CON	CON: 68	CON: 65.7	CON: 44 (64.7)		4.86±1.01
Chen 2023	China	CT: 12h a day for 7 days	CT: 40	CT 60.7 (14.2):	NR	VAS	6.64±1.66
		CPM: twice a day for 40-60	CPM: 40	CPM: 59.4 (15.6)			6.43±1.53

		min for 2 weeks					
Bruun-Olsen 2009	Norway	CPM: twice a day for 2 h for 1 week	CPM: 30	CPM: 68 (10)	CPM: 73	VAS	52±17
		CON	CON: 33	CON: 71 (10)	CON: 67		47±19
Chen 2013	China	CPM: 3 times a day for 2h for 4 days	CPM: 68	CPM: 69.2 (6.8)	NR	VAS	7.78±2.16
		CON	CON: 39	CON: 69.5 (8.1)			7.92±2.33
Can 2003	Turkey	CPM: once a day for 4-6h until discharge	CPM: 16	CPM: 61.6 (1.5)	CPM: 16 (100)	KSS	10.32±6.45
		CON	CON: 16	CON: 63.1 (1.7)	CON: 16 (100)		8±6.02
Gil-González 2022	Spain	CPM: 3 times a day for 2h until discharge	CPM: 105	CPM: 74.2 (6.8)	CPM: 38 (36)	VAS	6.4±1.9
		CON	CON: 115	CON: 73.3 (6.9)	CON: 45 (39)		6.5±1.2
Lenssen 2003	Netherlands	CPM: 4 h a day for 4 days	CPM: 20	CPM: 65 (9.2)	CPM: 15 (75)	NRS	3.6±2.8
		CON	CON: 20	CON: 65.7 (9.5)	CON: 12 (60)		3.8±2.7
Montgomery 1996	Sweden	CPM: 3 times a day for 3h until discharge	CPM: 28	CPM: 74 (5)	CPM: 24 (85.7)	VAS	7±2.25
		CON	CON: 32	CON: 76 (6)	CON: 24 (75)		8±2.25
Stasi 2020	Greece	CPM: twice a day for 60 min for 1 week	CPM: 25	CPM: 71.7 (6.2)	CPM: 18 (72)	VAS	5.64±2.1
		CON	CON: 25	CON: 72.6 (6.1)	CON: 22 (88)		6.12±2.24
Zhang 2023b	China	CPM: 3 times a day for 1-2h until discharage	CPM: 55	CPM: 51.8 (5.7)	CPM: 26 (47.3)	VAS	4.25±0.69
		CON	CON: 55	CON: 51.5 (5.7)	CON: 25 (45.5)		4.26±0.71

Note: CON: control; AP: acupuncture; EA: electroacupuncture; TENS: transcutaneous electrical nerve stimulation; NMES: Neuromuscular electrical stimulation; MLD: manual lymphatic drainage; KT: Kinesio taping; KT: CT: cryotherapy; CPM: continuous passive motion; VAS, the Visual Analog Scale; NRS, the Numeric Rating Scale; KSS, Knee Society Score; NR, not report.

Supplementary Table S7. Definition of physical therapy modalities

Nodes	Definition of physical therapy modalities
Physical therapy modalities	Therapeutic modalities frequently used in physical therapy specialty by physiotherapists to promote, maintain, or restore the physical and physiological well-being of an individual.
Acupuncture	The needles are inserted into the classical meridian, extra, painful, or trigger points.
Electroacupuncture	Electroacupuncture, which combines manual acupuncture with an electric stimulus, is a common treatment method for musculoskeletal diseases and injuries.
Transcutaneous electrical nerve stimulation	Transcutaneous electrical nerve stimulation is the noninvasive, transcutaneous, use of electrical stimulation to produce analgesia.
Neuromuscular electrical stimulation	Neuromuscular electrical stimulation consists of delivering preprogrammed trains of stimuli to muscles via surface electrodes positioned on the skin, with the ultimate goal to generate strong muscle contractions.
Manual lymphatic drainage	Manual lymphatic drainage is a light massage technique in a proximal to distal and then distal to proximal direction, following the lymphatic pathways.
Kinesio taping	An elastic adhesive tape used to support muscles and joints by mimicking the natural movement of the skin and improving circulation.
Cryotherapy	Cryotherapy involves the application of low temperatures to the skin surrounding the surgical site, by means of ice or cooled water, often delivered using specialized devices.
Continuous passive motion	Continuous passive motion is a way of providing regular movement to the knee using an external motorized device that passively moves the joint through a pre-set arc of motion.

Supplementary Table S8. The Pain rankings for different physical therapy modalities for sensitive analysis

Treatment	SUCRA(%)	PrBest (%)	Mean rank
CON	14.6	0.0	7.8
AP	48.6	6.1	5.1
EA	65.2	4.5	3.8
TENS	32.3	3.9	6.4
NMES	40.2	2.1	5.8
MLD	58.7	4.4	4.3
KT	84.3	34.1	2.3
CT	87.1	44.9	2.0
CPM	18.8	0.0	7.5

Note: CON: control; AP: acupuncture; EA: electroacupuncture; TENS: transcutaneous electrical nerve stimulation; NMES: neuromuscular electrical stimulation; MLD: manual lymphatic drainage; KT: kinesio taping; CT: cryotherapy; CPM: continuous passive motion.

Supplementary Table S9. The results of the network meta-regression

	β	SE	Z	P	95%CI
Participant age					
B vs CON	-0.324	0.192	-1.39	0.091	-0.699,0.052
C vs CON	-0.025	0.072	-0.35	0.725	-0.167,0.116
D vs CON	-0.053	0.228	-0.23	0.818	-0.500,0.395
E vs CON	-0.007	0.064	-0.12	0.907	-0.133,0.118
F vs CON	0.027	0.077	0.34	0.731	-0.125,0.178
G vs CON	0.016	0.034	0.48	0.628	-0.050,0.083
H vs CON	**0.164**	**0.048**	**3.45**	**0.001**	**0.071,0.257**
I vs CON	0.035	0.028	1.28	0.201	-0.019,0.090
Publication year					
B vs CON	-0.106	0.113	-0.94	0.348	-0.328,0.115
C vs CON	-0.068	0.071	-0.95	0.341	-0.207,0.072
D vs CON	-0.049	0.219	-0.22	0.823	-0.479,0.381
E vs CON	-0.049	0.073	-0.67	0.503	-0.193,0.095
F vs CON	**-0.165**	**0.067**	**-2.46**	**0.014**	**-0.297,-0.034**
G vs CON	0.222	0.197	1.13	0.260	-0.163,0.607
H vs CON	**-0.104**	**0.040**	**-2.62**	**0.009**	**-0.181,-0.026**
I vs CON	-0.011	0.022	-0.51	0.608	-0.053,0.031
Baseline pain (0-100)					
B vs CON	-0.039	0.029	-1.32	0.186	-0.097,0.019
C vs CON	-0.001	0.014	-0.10	0.917	-0.029,0.026
D vs CON	0.012	0.061	0.20	0.842	-0.107,0.131
E vs CON	-0.012	0.024	-0.48	0.628	-0.059,.0.036
F vs CON	-0.010	0.019	-0.55	0.581	-0.048,0.027
G vs CON	0.014	0.018	0.80	0.424	-0.021,0.049
H vs CON	-0.020	0.016	-1.22	0.224	-0.052,0.012
I vs CON	0.013	0.013	1.03	0.302	-0.012,0.039
Number of Interventions					
B vs CON	0.063	0.119	0.53	0.594	-0.159,0.296
C vs CON	0.049	0.050	0.97	0.330	-0.050,0.148
D vs CON	0.022	0.116	0.19	0.851	-0.206,0.249
E vs CON	0.008	0.018	0.47	0.638	-0.026,0.043
F vs CON	0.042	0.121	0.35	0.727	-0.195,0.280
G vs CON	-0.092	0.088	-1.05	0.294	-0.264,0.080
H vs CON	-0.034	0.028	-1.22	0.222	-0.088,0.020
I vs CON	0.014	0.046	0.31	0.758	-0.076,0.105
Time of Intervention Initiation					
B vs CON	-0.108	0.147	-0.74	0.462	-0.395,0.180
C vs CON	-0.393	0.354	-1.11	0.268	-1.087,0.302
E vs CON	-0.077	0.444	-0.17	0.862	-0.947,0.793
F vs CON	0.354	0.411	0.86	0.389	-0.452,1.159
G vs CON	0.030	0.430	0.07	0.945	-0.814,0.873
I vs CON	-0.078	0.389	-0.20	0.841	-0.841,0.685
Duration of Intervention					
B vs CON	0.056	0.083	0.68	0.496	-0.106,0.219
C vs CON	0.058	0.057	1.02	0.309	-0.054,0.171
D vs CON	0.022	0.116	0.19	0.852	-0.206,0.250
E vs CON	0.006	0.017	0.34	0.732	-0.028,0.040
F vs CON	-0.019	0.163	-0.12	0.906	-0.340,0.301
G vs CON	-0.088	0.086	-1.02	0.308	-0.257,0.081
H vs CON	-0.025	0.144	-0.18	0.861	-0.308,0.257
I vs CON	0.025	0.052	0.47	0.635	-0.077,0.126

Note: (CON) Control; (B) acupuncture; (C) electroacupuncture; (D) transcutaneous electrical nerve stimulation; (E) neuromuscular electrical stimulation; (F) manual lymphatic drainage; (G) Kinesio taping; (H) cryotherapy; (I) continuous passive motion.

Supplementary Figure S1. The box plots depicting effect modifiers for each treatment node and corresponding pairwise comparisons

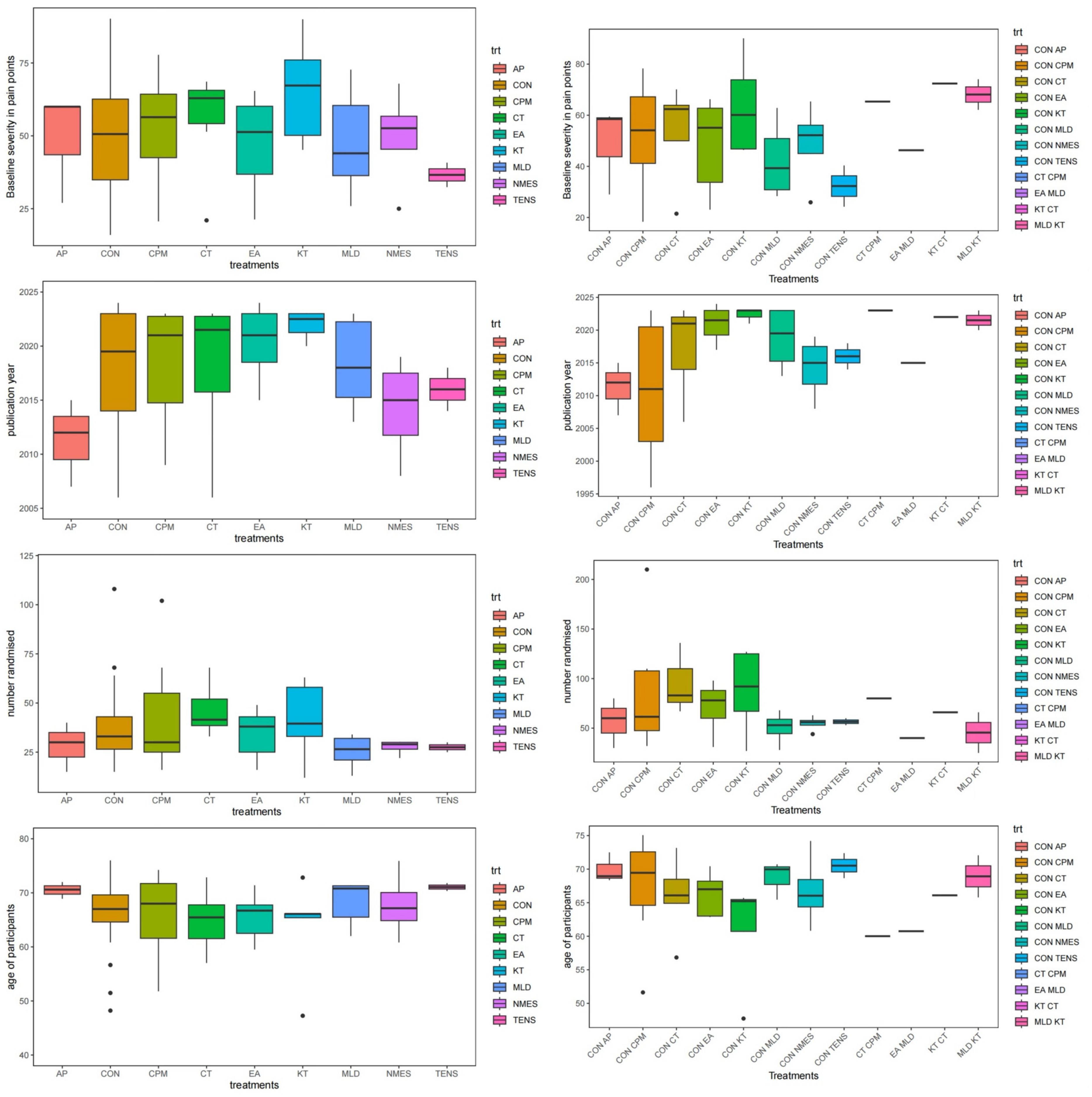

Note: CON: control; AP: acupuncture; EA: electroacupuncture; TENS: transcutaneous electrical nerve stimulation; NMES: neuromuscular electrical stimulation; MLD: manual lymphatic drainage; KT: kinesio taping; CT: cryotherapy; CPM: continuous passive motion.

Supplementary Figure S2. The risk of bias for each study

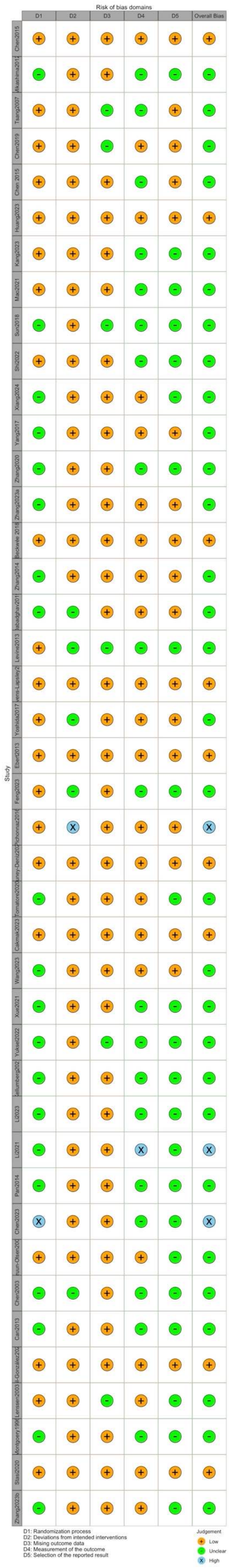

Supplementary Figure S3. The result of node-splitting method

```
. network sidesplit all, tau

Side    Direct                  Indirect                Difference                      tau
        Coef.      Std. Err.   Coef.      Std. Err.   Coef.      Std. Err.   P>|z|
A B            .           .           .           .           .           .       .           .
A C      -.57082    .2127657   -1.186246    .7642463    .6154264    .7932726  0.438    .6196037
A D            .           .           .           .           .           .       .           .
A E            .           .           .           .           .           .       .           .
A F   -.6087033    .3488626   -.4744654    .5100189   -.1342379    .6177766  0.828    .6259035
A G   -.8859099    .3025525   -.8568064    .6351081   -.0291036    .7034624  0.967     .626735
A H   -.8738079    .2980136   -1.429173    .6245414    .5553652    .6921263  0.422    .6169098
A I   -.1186321    .2315205     .899666    .7169249   -1.018298    .7533686  0.176    .6026318
C F    .5267501      .69846   -.0889746    .3762365    .6157246    .7933475  0.438    .6196059
F G    .0592649    .4933884   -.6501207    .4667126    .7093855    .6791819  0.296    .6162661
G H    .5781963    .6625765   -.3719498    .4256059    .9501461    .7874799  0.228    .6137321
H I    1.728318    .6580375    .7100036    .3669344    1.018315    .7534283  0.177    .6026331
```

Note: (A) Control; (B) acupuncture; (C) electroacupuncture; (D) transcutaneous electrical nerve stimulation; (E) neuromuscular electrical stimulation; (F) manual lymphatic drainage; (G) Kinesio taping; (H) cryotherapy; (I) continuous passive motion.

Supplementary Figure S4. The prediction interval plot for pain network

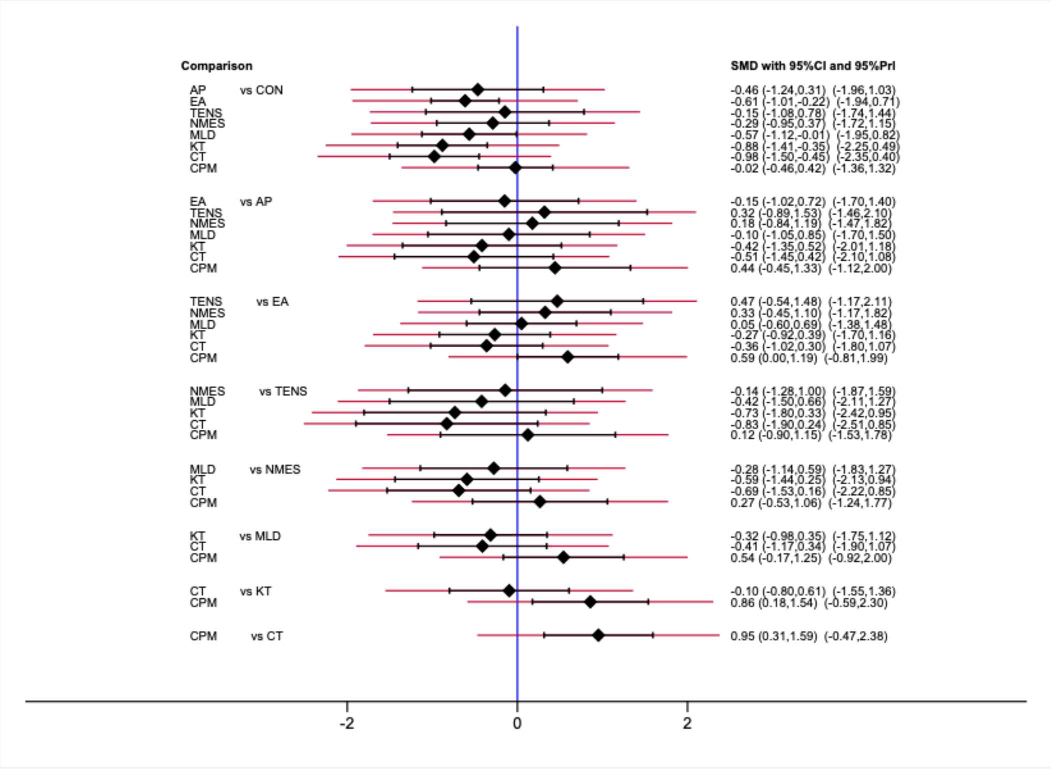

Note: CON: control; AP: acupuncture; EA: electroacupuncture; TENS: transcutaneous electrical nerve stimulation; NMES: Neuromuscular electrical stimulation; MLD: manual lymphatic drainage; KT: Kinesio taping; KT: CT: cryotherapy; CPM: continuous passive motion; VAS, the Visual Analog Scale; NRS, the Numeric Rating Scale; KSS, Knee Society Score; NR, not report.

Supplementary Figure S5. The comparison-adjusted funnel plots for pain network

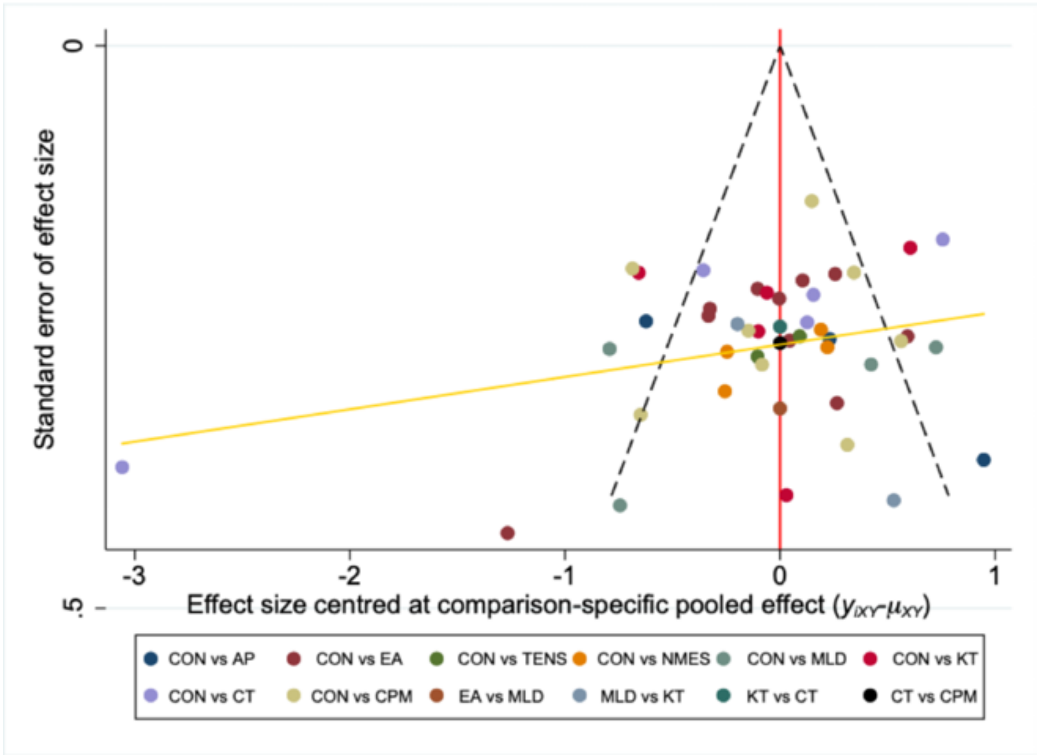

Note: CON: control; AP: acupuncture; EA: electroacupuncture; TENS: transcutaneous electrical nerve stimulation; NMES: neuromuscular electrical stimulation; MLD: manual lymphatic drainage; KT: kinesio taping; KT: CT: cryotherapy; CPM: continuous passive motion.

Supplementary Figure 6. The prediction interval plot for sensitive analysis

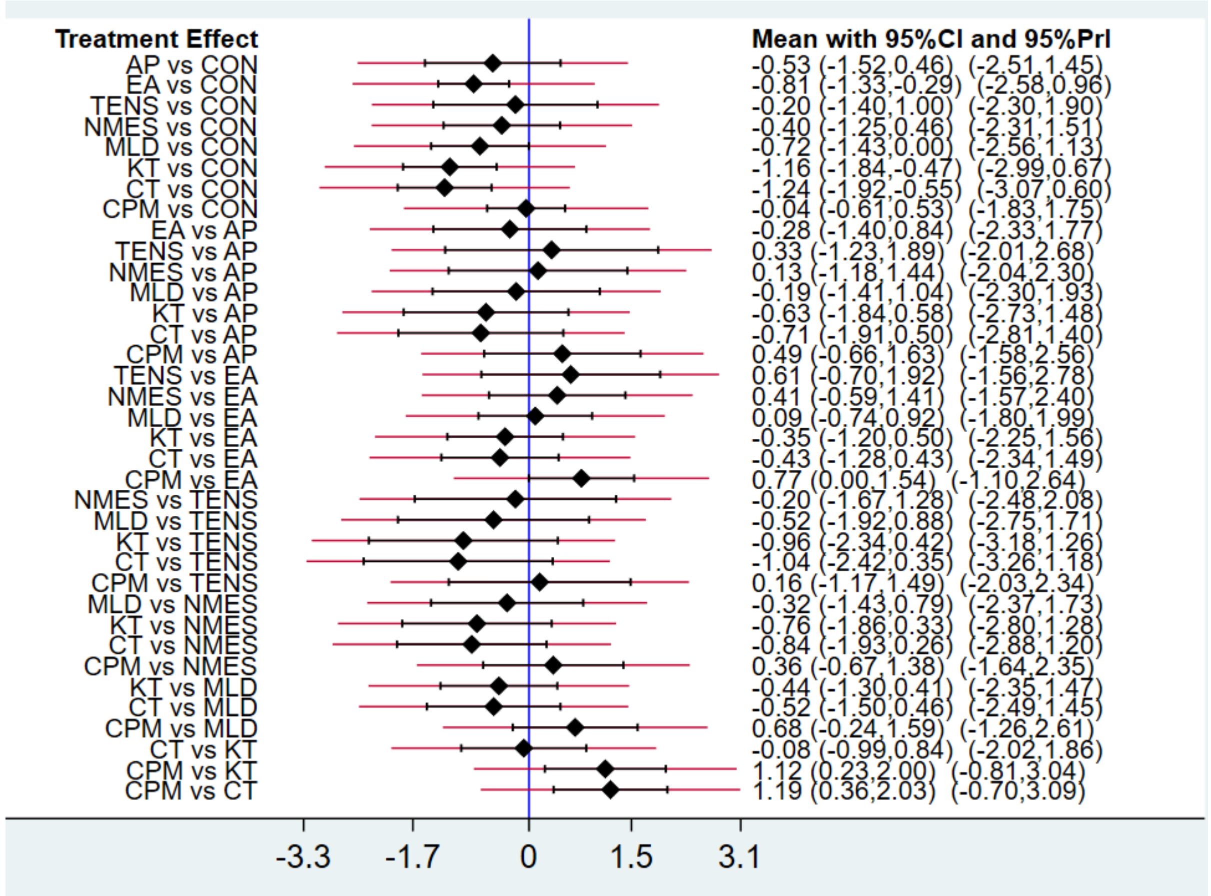

Note: CON: control; AP: acupuncture; EA: electroacupuncture; TENS: transcutaneous electrical nerve stimulation; NMES: neuromuscular electrical stimulation; MLD: manual lymphatic drainage; KT: kinesio taping; CT: cryotherapy; CPM: continuous passive motion. Global inconsistency tests: chi2(7) = 3.87 Prob > chi2 = 0.7941
